# Field and in-silico analysis of harvest index variability in maize silage

**DOI:** 10.3389/fpls.2023.1206535

**Published:** 2023-06-19

**Authors:** Jonathan Jesus Ojeda, M. Rafiq Islam, Martin Correa-Luna, Juan Ignacio Gargiulo, Cameron Edward Fisher Clark, Diego Hernán Rotili, Sergio Carlos Garcia

**Affiliations:** ^1^ Centre for Sustainable Agricultural Systems, University of Southern Queensland, Toowoomba, QLD, Australia; ^2^ Tasmanian Institute of Agriculture, University of Tasmania, Hobart, TAS, Australia; ^3^ Dairy Science Group, School of Life and Environmental Sciences, Faculty of Science, The University of Sydney, Camden, NSW, Australia; ^4^ NSW Department of Primary Industries, Menangle, NSW, Australia; ^5^ Livestock Production and Welfare Group, School of Life and Environmental Sciences, Faculty of Science, The University of Sydney, Camden, NSW, Australia; ^6^ Cátedra de Cerealicultura, Departamento de Producción Vegetal, Facultad de Agronomía, Universidad de Buenos Aires, Buenos Aires, Argentina; ^7^ Instituto de Investigaciones Fisiológicas y Ecológicas Vinculadas a la Agricultura (IFEVA) Facultad de Agronomía, Universidad de Buenos Aires, Consejo Nacional de Investigaciones Científicas y Técnicas (CONICET), Buenos Aires, Argentina

**Keywords:** silage quality, APSIM, crop modelling, calibration, forage, *Zea mays* L.

## Abstract

Maize silage is a key component of feed rations in dairy systems due to its high forage and grain yield, water use efficiency, and energy content. However, maize silage nutritive value can be compromised by in-season changes during crop development due to changes in plant partitioning between grain and other biomass fractions. The partitioning to grain (harvest index, HI) is affected by the interactions between genotype (G) × environment (E) × management (M). Thus, modelling tools could assist in accurately predicting changes during the in-season crop partitioning and composition and, from these, the HI of maize silage. Our objectives were to (i) identify the main drivers of grain yield and HI variability, (ii) calibrate the Agricultural Production Systems Simulator (APSIM) to estimate crop growth, development, and plant partitioning using detailed experimental field data, and (iii) explore the main sources of HI variance in a wide range of G × E × M combinations. Nitrogen (N) rates, sowing date, harvest date, plant density, irrigation rates, and genotype data were used from four field experiments to assess the main drivers of HI variability and to calibrate the maize crop module in APSIM. Then, the model was run for a complete range of G × E × M combinations across 50 years. Experimental data demonstrated that the main drivers of observed HI variability were genotype and water status. The model accurately simulated phenology [leaf number and canopy green cover; Concordance Correlation Coefficient (CCC)=0.79-0.97, and Root Mean Square Percentage Error (RMSPE)=13%] and crop growth (total aboveground biomass, grain + cob, leaf, and stover weight; CCC=0.86-0.94 and RMSPE=23-39%). In addition, for HI, CCC was high (0.78) with an RMSPE of 12%. The long-term scenario analysis exercise showed that genotype and N rate contributed to 44% and 36% of the HI variance. Our study demonstrated that APSIM is a suitable tool to estimate maize HI as one potential proxy of silage quality. The calibrated APSIM model can now be used to compare the inter-annual variability of HI for maize forage crops based on G × E × M interactions. Therefore, the model provides new knowledge to (potentially) improve maize silage nutritive value and aid genotype selection and harvest timing decision-making.

## 1 Introduction

Maize (*Zea mays* L.) silage is a key component of the dairy cow feed ration in intensive dairy production systems and is also used in temperate pasture-based systems to supplement cows’ diets when pasture availability is low (Wales and Kolver, 2017). The inclusion of maize in dairy cows’ diets has increased over the past 30 years due to its high total yield (Ferraretto et al., 2018), digestible energy content (Sucu et al., 2016), and the opportunity for relatively long-term storage as silage with limited nutritive value loss when ensiled properly (Borreani et al., 2018).

Crop growth, phenology, and yield are also affected by genetics (Barlow et al., 2012; Borreani et al., 2018; Gruber et al., 2018), environmental (Bernardes et al., 2018), and management factors (Nilahyane et al., 2020; Petković et al., 2022). For the latter, the inputs of nitrogen (N) fertiliser and water have the greatest economic and environmental impact with regards to maize production (Petković et al., 2022). Optimal irrigation of maize crops can increase biomass partitioning to grain (Li et al., 2020) and the management of irrigation and N (defined by rate and timing) can affect dry matter allocation to different parts of the plant, and, therefore, maize silage nutritive value (Nilahyane et al., 2020; Zhou et al., 2021). Maize silage of high quality can result in a feed ingredient with a high recovery of dry matter, energy, and highly digestible nutrients compared with the fresh crop (Kung et al., 2018). More specifically, starch content and, therefore, grain yield can markedly improve silage forage quality (Nazli et al., 2019) and, consequently, milk production of dairy systems (Boerman et al., 2015).

However, maize yield (grain and total plant) and climatic conditions can vary greatly both spatially and temporally (Khalili et al., 2013; Nilahyane et al., 2020). Thus, predictive tools that provide a quantification of the variability in the grain proportion relative to biomass, i.e., the ratio between grain yield and aboveground biomass (HI) of maize crops grown for silage, are urgently required. Such tools would allow farmers and advisors to predict the inter-annual variability of maize HI based on genotype (G) × environment (E) × management (M) interactions and, therefore, provide a decision support system to aid genotype selection (Hütsch and Schubert, 2017; Gruber et al., 2018) and harvest timing (Borreani et al., 2018; Ferraretto et al., 2018) decision making for optimising ration formulations. Mechanistic crop models (hereafter ‘models’) are effective tools for understanding the complexity of system interactions to achieve productivity and environmental goals (Archontoulis et al., 2014). Models enable the analysis of climate variability on crop growth and thus inform the development of adaptation strategies (Challinor et al., 2013; Jahangirlou et al., 2023). Despite the increasing use of models, there are implicit model uncertainties (i.e., deviations derived from a probability distribution of simulations generated using different parameters) and prediction uncertainties (i.e., deviations between simulations and observations commonly named residuals) (Chapagain et al., 2022). Therefore, there is a need for a standardised approach to quantify both model and prediction uncertainty (Chapagain et al., 2022) to increase model accuracy.

The Agricultural Production Systems Simulator (APSIM) (Holzworth et al., 2014) is a mechanistic biophysical crop model that estimates crop growth and development in response to G × E × M interactions. This model has been widely used for maize under several productive scenarios (Archontoulis et al., 2014; Ojeda et al., 2018b; Rotili et al., 2020; Kamali et al., 2022; Jahangirlou et al., 2023). These studies demonstrated that APSIM had a reasonable to very good accuracy for biomass and maize grain yield estimations. Apart from some exceptions (Pembleton et al., 2013; Teixeira et al., 2017; Ojeda et al., 2018a; Ojeda et al., 2018b; Morel et al., 2020), previous studies assessing the performance of APSIM to estimate crop yield have been largely focused on maize crops grown for grain, using specific grain yield harvest-destined genotypes and crop management to achieve high grain yields. While these studies mainly assessed the effect of crop management and environment on maize biomass and grain yield, the effect of genotype on HI has not been assessed under a wide range of crop management conditions. There is only one study where maize quality was estimated by combining APSIM yield simulations with logistic regression models with a focus on grain composition although it used grain genotypes and generated predictions based on simulated grain dry weight (Jahangirlou et al., 2023). The model’s ability to estimate crop growth and phenological responses for maize silage genotypes and management under contrasting environments and focus on the prediction of HI across a wide range of G × E × M is still required.

The objectives of this study were to (i) identify the main drivers of grain yield and HI variability, (ii) calibrate APSIM to estimate crop growth, development, and plant partitioning using detailed experimental field data, and (iii) explore the primary sources of HI variability of silage maize in a wide range of G×E×M combinations.

## 2 Material and methods

In this study, we applied two levels of analysis which are described in detail in 2.1 and 2.5. First, we identified the main drivers of HI (ratio between grain yield and biomass) variability at plot level using a non-complete factorial combination of fields which was analysed by experiment depending on the factors present in each experiment. Second, we used APSIM to generate results from a complete factorial combination (and therefore interactions) where we used a widely known Analysis of Variance approach to decompose the effect of each factor and their interactions on HI variability. For the analysis of simulated data, we applied a G × E × M model to disentangle the effect of G, E, and M on HI variability at one site. The E component was implicit in the inter-annual variability of climate across years.

### 2.1 Experimental datasets

Data from four field experiments conducted by the University of Sydney in different locations (P-farm, Westwood, and Mayfarm) near Camden, NSW, Australia (33° 59’ 53.52” S; 150° 36’ 45.72” E) were used to determine the key drivers of grain yield and HI variability and to calibrate the maize crop module in APSIM Classic (v7.10; https://www.apsim.info/) to predict biomass partitioning (**Table 1**). The Mayfarm site included two experimental years (MayfarmY1 and MayfarmY2) and several in-season crop measurements. A detailed description of these two experiments is provided by Islam et al. (2011); Islam et al. (2012) and Islam and Garcia (Islam and Garcia, 2012; Islam and Garcia, 2014). P-farm and Westwood sites included one experiment each and data at the final harvest for silage. For P-farm, hybrid forage maize (Pioneer 2307) was sown on 4 November 2012 in 20 × 20 m blocks within a 30-ha paddock. Maize was planted at two plant densities (6.6 plants m^-2^ and 7.4 plants m^-2^) with row-to-row distances of 79 cm and harvested at three different stages of maturity [31%, 38%, and 45% whole plant dry matter (DM)] on 14 March, 27 March, and 8 April in 2013, respectively, for both sowing densities. Each treatment (density × maturity stage at harvest) was replicated in three blocks. For Westwood, two hybrid forage maize (PacificX and PioneerX) were grown in 60 × 15 m blocks within a 20-ha paddock. Both maize forages were sown on 20 November 2012 at two plant densities (5.5 plants m^-2^ and 8.2 plants m^-2^) with row-to-row distances of 68 cm and harvested at three different stages of maturity (31%, 42%, and 45% DM) on 20 March, 8 April, and 15 April in 2013, respectively, for both sowing densities. In both P-farm and Westwood, each treatment was assigned to a plot of 5 × 3 m and 5 × 2 m, respectively, with a 1 m buffer on each side of the plot, and plots were randomised within each block. All experiments included combinations of different factors (crop management and genotypes) as shown in **Table 1**. For all experiments, total aboveground biomass and its partitioning between plant organs (leaf weight, stem weight, and grain + cob weight) were measured. Total biomass was measured by harvesting the whole plot, while leaf, stem, and grain + cob weight were estimated based on the proportions determined from one sampled plant per plot (Islam et al., 2012; Islam and Garcia, 2014). For the Mayfarm site, leaf number and normalised difference vegetation index (NDVI) were also measured sequentially over the whole growing period. The number of observations varied between experiments and variables assessed (**Table 2**). The NDVI observations were converted to the proportion of intercepted photosynthetically active solar radiation, commonly known as fPAR or cover green in APSIM, using the equation proposed by Pellegrini et al. (2020) as follows:

**Table 1:** Description of maize field experiments, factors of analysis, treatments, and observations (in-season and at final harvest) used to calibrate the Agricultural Production Systems Simulator.

Experiments	P-farm		Westwood		MayfarmY1		MayfarmY2	
	*Factor*	*Value*	*Factor*	*Value*	*Factor*	*Value*	*Factor*	*Value*
**Factorial combinations**	Harvest date	120 DAS*	Harvest date	120 DAS	Sowing date	20-Oct	Irrigation rates	0 mm (0%)
		133 DAS		139 DAS		3-Nov		153 mm (33%)
		145 DAS		141 DAS	N fertilisation rate pre-sowing	0 kgN ha^-1^		305 mm (66%)
	Sowing density	6.3-6.9 plants m^-2^	Genotype	PioneerX		135 kgN ha^-1^		480 mm (100%)
		7.2-7.7 plants m^-2^		PacificX	N fertilisation rate post-sowing	0 kgN ha^-1^	N fertilisation rate pre-sowing	0 kgN ha^-1^
			Sowing density	5.2-6 plants m^-2^		79 kgN ha^-1^		135 kgN ha^-1^
				6.4-9.3 plants m^-2^		158 kgN ha^-1^	N fertilisation rate post-sowing	0 kgN ha^-1^
								79 kgN ha^-1^
								158 kgN ha^-1^
**Treatments (replications)**	6 (3)		12 (4)		12 (4)		24 (4)	
**Obs. variables in-season by treatment**					12 (2)		24 (2)	
**Obs. Variables at harvest by treatment**	6 (3)		12 (4)		12 (4)		24 (4)	

*DAS, days after sowing.

**Table 2:** The number of observations per variable (in-season and at final harvest) by experiment.

	P-farm	Westwood	MayfarmY1	MayfarmY2	Total
Leaf number			54	144	198
Canopy green cover			54		54
Aboveground biomass weight	6	12	64	96	178
Grain/cob weight	6	12	64	96	178
Stover weight	6	12	64	96	178
Leaf weight	6	12	64	168	250
Stem weight	6	12	64	144	226
Grain number	6	12	12		30
Grain size	6	12	12		30


Eq. (1) Green Cover=(1.5 × NDVI)−0.29

### 2.2 Model description

We used the maize crop module within APSIM Classic 7.10 (Holzworth et al., 2014). The model has been described at https://www.apsim.info/documentation/model-documentation/crop-module-documentation/maize/ and calibrated with satisfactory results across an extensive range of environments (Ojeda et al., 2018b; Wu et al., 2021). A complete description of the model structure and parameters was provided by Brown et al. (2014a) and Brown et al. (2019). The model contains algorithms that simulate crop phenology, growth, and soil-plant C and N dynamics. In brief, crop phenology is simulated using thermal time thresholds for each phenological stage. The model accumulates thermal time until a target is achieved, which defines the change of the stage. The growing period for the whole crop cycle of maize in APSIM is mainly influenced by the cumulative thermal time from emergence to the end of the juvenile phase and flowering (R1; Ritchie et al., 1996) to physiological maturity (R6), as well as by the phyllochron (leaf appearance rate). When water and N conditions are optimum, crop daily growth rate is only limited by photosynthetically active solar radiation (PAR) and is calculated as the product of intercepted PAR and radiation use efficiency. When the crop is under water stress, biomass accumulation is calculated as the product of potential crop transpiration (limited by available soil moisture within field capacity and permanent wilting point, root extent, and water uptake capacity) and transpiration efficiency, adjusted for atmospheric vapour pressure deficit (Brown et al., 2014b). **Table S1** shows details on key embedded processes in the APSIM-Maize model.

Potential biomass partitioning among plant parts in APSIM depends on the crop development stage and uses partitioning ratios (Brown et al., 2019). Between emergence and flag leaf (the last leaf to appear and expand), the fraction of biomass that is provisionally allocated to the growing leaves decreases as the number of fully expanded leaves increases. Between tassel initiation and flag leaf, the biomass remaining after allocation to the leaves is partitioned between the stem and the developing cobs with a fixed ratio. After flag leaf, biomass is partitioned between the stem and the cobs only, until partitioning to the grain starts at the onset of grain filling (Soufizadeh et al., 2018). Grain yield is calculated as the product of grain number and grain size. Grain number is estimated from the average daily growth rate per plant during a thermal time window defined as the critical period, generally starting at 227°Cd before flowering (Otegui and Bonhomme, 1998) and finishing at the start of grain filling and the potential grain number per cob (Liu et al., 2022). Total grain assimilate demand is the product of grain number and a potential grain growth rate, which is based on potential grain size and grain filling duration. A detailed description of the association between potential grain number and grain size has been described by Gambín et al. (2006).

### 2.3 Simulation configuration

#### 2.3.1 Climate and soil

Experiment geolocations were used to retrieve climate and soil data to calibrate the model. Historical daily climate data (daily rainfall, maximum and minimum air temperature, and global solar radiation) from SILO (https://www.longpaddock.qld.gov.au/silo/point-data/) were used as inputs to the model. This daily interpolated climate dataset has been widely tested with weather station data across Australia (Jeffrey et al., 2001; Beesley et al., 2009) and also used for crop modelling purposes (Ojeda et al., 2020). Soil data were extracted from the Soil and Landscape Grid of Australia Database, which generates a synthetic APSIM soil derived from pedotransfer functions (https://www.asris.csiro.au/ASRISApi#!/APSIM32Services/ApsoilFromGrid_getApSoilTypeMap). Due to the high spatial proximity of the experiments and after consultation with the experimentalists about the soil types of the experiments, we decided to use a single soil profile for the model setup in all experiments. A complete description of the soil parameters used to configure the soil profile in APSIM can be found in **Table S2**.

#### 2.3.2 Crop management

Actual crop management practices implemented in the field were used to parametrise the crop management settings in the model for in-silico experiments. Sowing density varied among experiments from 5.5 to 11.4 plants m^-2^, and the distance between rows was the same for all experiments (680 mm) except for P-farm (790 mm). Sowing and harvest date varied among experiments although all crops were harvested for silage purposes (i.e., at a whole plant DM content of 21-47%; **Table S3**). In all simulations, a harvesting rule was set to remove the biomass at a height of 150 mm as per standard practice in commercial farms. We used two model scripts to configure the actual N fertilisation rates of the field experiments: one at the sowing date and the other at V6. Nitrogen fertilisation was applied as urea in all experiments and the rates varied from 0 to 293 kg N ha^-1^ (**Table S3**). The irrigation module was parametrised to mimic the actual irrigation amounts applied in the field. A detailed description of the crop management practices used to set up the model can be found in **Table S3**.

#### 2.3.3 Genotypic parametrisation

There are more than 90 maize genotypes available in APSIM. Therefore, the computing cost to test the accuracy of the model using all these APSIM default genotypes against our experimental data would be too high and impossible to implement. In addition, all hybrids used in the experiments employed in this study were silage genotypic types unavailable in the APSIM cultivar library. Thus, the parameterisation of new genotypes was required. We classified the genotypic parameters into parameters for parametrisation for optimisation or default model parameters (**Table S4**). This classification helped us to identify the calibration method to be implemented for each group of parameters. A *parameter for parametrisation* can be calibrated using field observations, while a *parameter for optimisation* is uncertain and, therefore, requires to be optimised. We defined a *default parameter* when there was a lack of data available to include them in the optimisation procedure and therefore the original genotypic parameter in APSIM was used for that parameter. The genotypic parameter values after calibrating the model can be found in **Table 3**.

**Table 3:** Description of calibrated genotypical parameters used in the Agricultural Production Systems Simulator by experiment.

	P-farm	Westwood		MayfarmY1	MayfarmY2	
Genotype	Pioneer 2307	PacificX	PioneerX	PioneerY	Pioneer 31H50	
Parameter	value					unit
*aX0*	0.57	0.61	0.67	0.67	0.67	proportion
*largestLeafParams*	1666 -1.17 0.047	1000-1.17 0.047	1200 -1.17 0.047	3400 -1.17 0.047	3050 -1.17 0.047	–
*leaf_app_r1*	65	65	65	85	90	°Cd
*leaf_app_r2*	36	36	36	45	45	°Cd
*leaf_app_r3*	36	36	36	45	45	°Cd
*leaf_no_rate_ch1*	8	8	8	8	9	leaves
*leaf_no_rate_ch2*	3	3	3	3	6	leaves
*ph1*	12.5	12.5	12.5	12.5	12.5	h
*ph2*	20	20	20	20	20	h
*ph_s*	0	0	0	0	0	h
*tt_emerg_to_endjuv*	310	350	330	230	270	°Cd
*tt_flag_to_flower*	50	50	50	50	50	
*tt_flower_to_start_grain*	170	170	170	170	170	°Cd
*tt_flower_to_maturity*	800	600	1000	700	600	°Cd
*tt_maturity_to_ripe*	1	1	1	1	1	°Cd
*frac_stem2flower*	0.26	0.46	0.12	0.47	0.55	0-1
*stem_trans_frac*	0.61	0.4	0.4	0.26	0.275	0-1
*potKernelWt*	315	332	311	319	315	mg grain^-1^
*GNk*	0.33	0.38	0.366	0.354	0.321	–
*GNmaxCoef*	323	339	309.5	350	297	grains plant^-1^

#### 2.3.4 Optimisation procedure

**Figure 1:**
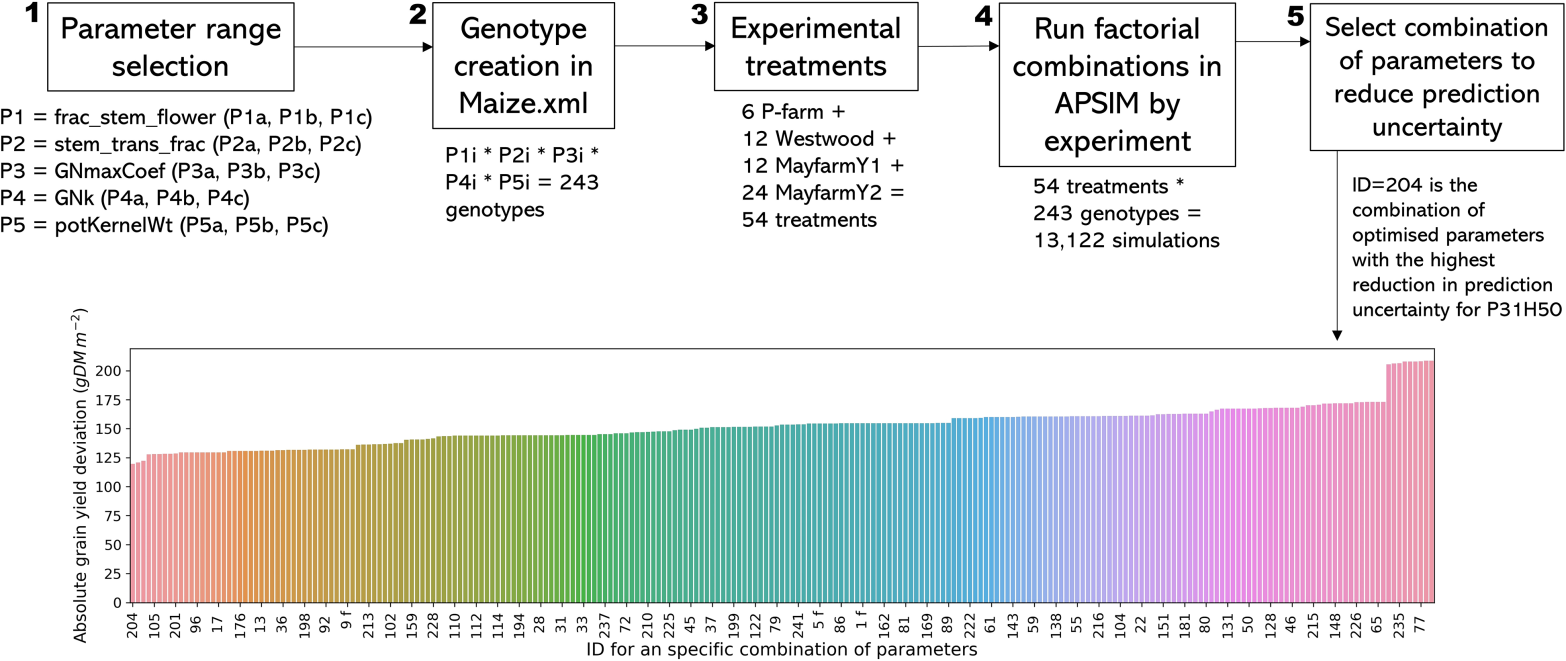
Optimisation process description for genotype parameters. Steps 1-4 apply for all genotypes, and step 5 and the bar plot show a specific example for MayfarmY2 for genotype Pioneer 31H50. Subscript letters in steps 1 and 2 indicate each level of a given parameter (i.e., for P1a, a = 0.4). Subscript ‘i’ in step 2 represents ‘a’, ‘b’, or ‘c’ from step 1. Labels in the x-axis represent one ID combination of every four possible combinations for display purposes.

We carried out a multi-step optimisation procedure to select the best combination of values for the most sensitive genotypic parameters (**Figure 1**). We followed a series of steps: *Step 1)* We generated a logical range of possible values of parameters for optimisation based on the literature. We used the wide range of maize parameters reported by Rotili et al. (2020) based on in-farm and on-research station trials conducted across New South Wales and Queensland, Australia, over three seasons. *Step 2)* We created artificial genotypes in the Maize.xml plant module in APSIM using all combinations of parameters previously selected. *Step 3)* We differentiated the experimental treatments by genotype used. *Step 4)* We ran APSIM using a range of values for five parameters that affect biomass partitioning to grain: *frac_stem_flower* (0.05, 0.4, and 0.75), *stem_trans_frac* (0.05, 0.4, and 0.75), *GNk* (0.27, 0.33, and 0.39), *GNmaxCoef* (215, 282.5, and 350 grains plant^-1^), and *potKernelWt* (299, 319, and 339 mg grain^-1^) (**Table S4**). *Step 5)* We selected the set of parameters that optimised the grain yield simulations, i.e., the highest reduction in the prediction uncertainty (simulated grain yield – observed grain yield) for this model output at the experiment level (one genotype by experiment).

### 2.4 Data analysis, calculations, and model evaluation

First, we curated and formatted the data available from the four above-mentioned experiments to unify units and names before implementing the APSIM modelling calibration. Data curation and analysis were done using Python 3.8.12 (https://www.python.org) through the Jupyter Lab interface (https://jupyter.org/). The code developed during this procedure is hosted in a private GitHub repository (https://github.com/Jjguri/DairyUp) (access to the repository can be provided by request to the corresponding author). We used boxplots and scatter plots to investigate relationships and general trends of grain yield, HI, and biomass partitioning. In this paper, we considered HI as the ratio between grain yield and aboveground biomass independent of the maize growth stage when the measurements were collected (considering mainly differences in the percentage of DM for silage purposes). The correlation between observed HI and plant components was assessed for all experiments (**Figure S1**). This analysis allowed us to identify the main causes of variance for observed data.

Second, we calculated statistical coefficients, including the Root Mean Square Error (RMSE) and the Root Mean Square Percentage Error (RMSPE), the Nash–Sutcliffe model efficiency coefficient (NSE), and Lin’s Concordance Correlation Coefficient (CCC). The RMSE and RMSPE provide a quantification of the differences between simulated and observed values in the unit of the variable and percentage, respectively. On the other hand, the NSE is a normalised statistic that determines the relative magnitude of the residual variance compared to the observed data variance. The CCC integrates precision through Pearson’s correlation coefficient, which represents the proportion of the total variance in the observed data that can be explained by the model and accuracy by a bias which indicates how far the regression line deviates from the concordance (y=x) line. All statistical indicators are suitable to evaluate model performance; however, CCC integrates model precision and accuracy, and the evaluation can be interpreted by assessing a single number from -1 to 1. Following the approach proposed by Ojeda et al. (2017), we created a categorical variable to evaluate the model performance based on CCC. The model performance (defined as the sum of accuracy and precision) to simulate all variables was evaluated as: “very good” when CCC ≥ 0.90, “satisfactory” when 0.8 ≤ CCC< 0.9, “acceptable” when 0.4 ≤ CCC< 0.8, and “poor” for other values (Tedeschi, 2006). We used the *NumPy* and *pandas* packages in Python to assess the model prediction uncertainty (simulated values – observed values). The statistical coefficients were used to create a heatmap of global model performance for the variables described in **Table 2**. Additionally, we visually compared observed vs. simulated state variables by experiment for each observation date (in-season and final harvest).

### 2.5 Scenario analysis of harvest index variance

To analyse the variance of HI across a wide range of scenarios, we ran APSIM with the calibrated and validated genotypes across a 50-year period (1972-2021) for several combinations of crop management factors (described below). We used climate and soil information from the Mayfarm site to run the long-term simulations. We combined the effect of irrigation (rainfed, deficit, and well-irrigated), sowing date (20 Oct and 3 Nov), sowing density (5.2, 7.2, and 9.3 pl m^-2^), N application rate (0, 125, and 250 kg N ha^-1^), genotype [early (PacificX; 1170°Cd from emergence to physiological maturity) and late (PioneerX; 1550°Cd from emergence to physiological maturity)], and harvest date (125, 138, and 150 days after sowing). The levels of all factors were defined based on the data range of the field experimental data used for the calibration. Three irrigation levels were defined based on the ratio between actual soil water and soil field capacity (0, rainfed; 0.4, deficit irrigation; 0.8, well irrigated). The model quantified this ratio every day and applied irrigation if the ratio exceeded the indicated thresholds for each treatment. In total, we generated 324 scenarios (three irrigations × two sowing dates × three sowing densities × three N rates × three harvest dates × two genotypes) across 50 years (16,200 scenario x year combinations). The main drivers of HI variability were assessed through boxplots and analysis of variance.

To determine the contribution of each factor to the total HI variability, variance-based sensitivity indices were computed (Monod et al., 2006; Kamali et al., 2022). According to this method, the variance of the model output is decomposed into fractions that can be attributed to various factors. These methods measure the sensitivity of each factor independently and also quantify the effect of interactions. Two indices, namely, Main Effect ( ME ) (Eq. 2) and Total Effect (*TE*) (Eq. 3), were used to disentangle the variance caused by one source from the variance caused by the interaction.


(2) $$ME_{i}=\frac{Variance\left(E\left[HI\;X_{i}\right]\right)}{variance\;\left(Y\right)}$$

And


(3) $$TE_{i}=1-\;\frac{Variance\;\left(E\left[HI\;X_{-i}\right]\right)}{Variance\;\left(Y\right)}$$

where $E\left[HI\;X_{i}\right]$ denotes the expected value of HI across all sources $X_{i}$ (irrigation, sowing date, sowing density, N rate, harvest date, and genotype), while $E\left[HI\;X_{-i}\right]$ is the expected value of HI across all sources except for $X_{i}$ . In other words, *ME* explains the share of the components to HI variability without interactions, i.e., if ME=1 , the assessed factors explain the entire proportion of HI variability, but if M<1 , residuals exist, which means additional factors are required to explain this variability. *TE* represents the interaction of a given factor with other factors, i.e., high *TE* values for a given factor denote high interactions of that factor with other factors; therefore, *TE* does not include residuals.

## 3 Results

### 3.1 Drivers of observed harvest index variability

**Figure 2:**
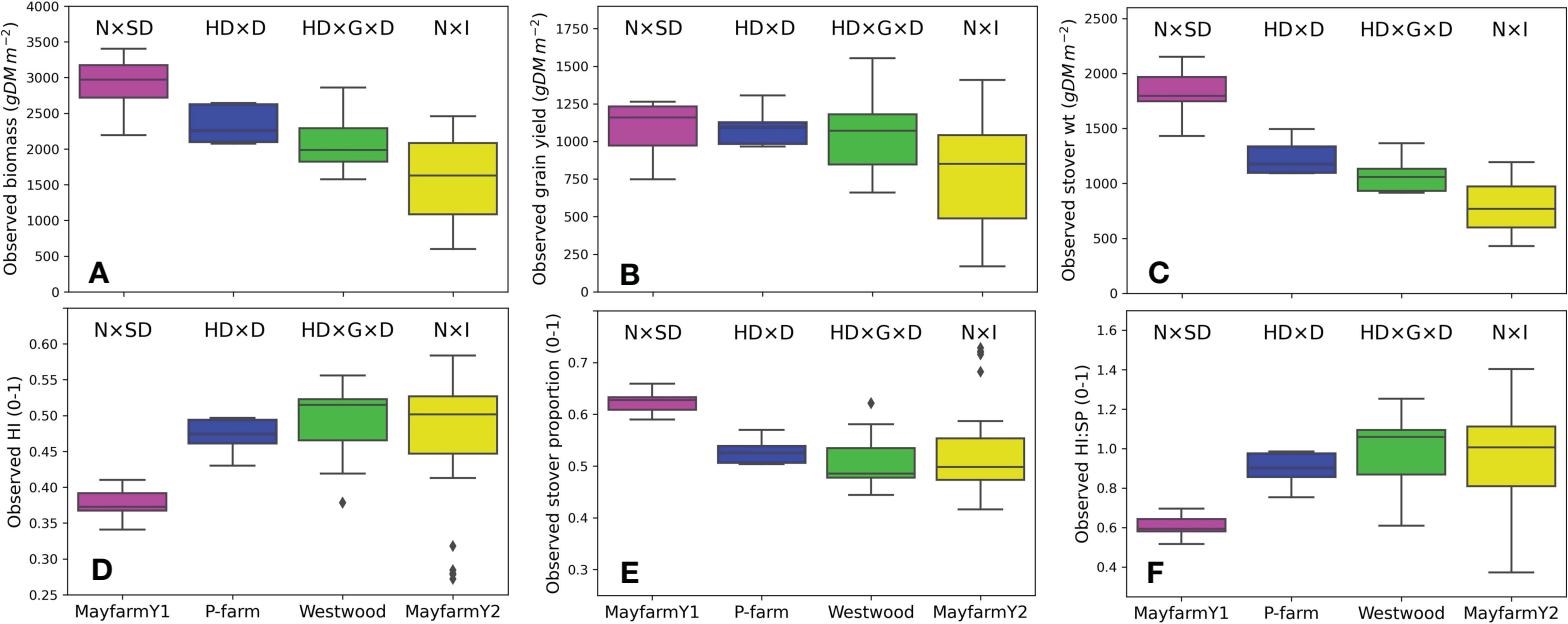
Observed **(A)** aboveground biomass, **(B)** grain yield, **(C)** stover weight, **(D)** harvest index (HI), **(E)** stover proportion (SP; stover weight/aboveground biomass), and **(F)** harvest index:stover proportion (HI : SP) by experiment. The letters above each boxplot indicate the combinations of factors assessed in each experiment. N, N fertilisation rate; SD, sowing date; HD, harvest date; D, plant density; G, genotype; I, irrigation rate. Grain yield data in **(B)** include only final harvest measurements (i.e., it does not include cob weight measurements).

Observed biomass and grain yield varied across experiments depending on the genotype and crop management. On average, the observed biomass ranged from 1,558 g DM m^-2^ (MayfarmY2) to 2,922 g DM m^-2^ (MayfarmY1). Grain yield ranged from 772 g DM m^-2^ (MayfarmY2) to 1,096 g DM m^-2^ (MayfarmY1) and stover weight ranged from 786 g DM m^-2^ (MayfarmY2) to 1,826 g DM m^-2^ (MayfarmY1). While the average observed HI was relatively similar (0.472 to 0.487) between P-farm, Westwood, and MayfarmY2, it was considerably lower on MayfarmY1 (0.374). Consequently, we found the highest stover proportion at MayfarmY1 (0.626) (**Figure 2**).

In the experiments with the highest plant densities (MayfarmY1 and MayfarmY2), observed HI was significantly correlated with observed grain yield. However, it was only associated with aboveground biomass and stover weight at MayfarmY2 (**Figure S1**; **Table 4**). The associations were mainly affected by irrigation rate (i.e., water crop status) and the genotype used in the experiments. While HI considerably increased when the irrigation rate increased up to 66% at MayfarmY2, it remained relatively constant when irrigation rates surpassed this threshold (**Figure S1**). A complete description of the relationship between observed HI and aboveground biomass, stover, and grain weight is provided in **Figure S1**.

**Table 4:** The statistical description of correlations between grain yield and harvest index (HI) vs. aboveground biomass, grain yield, and stover weight in each experiment.

Experiment	Dependent Variable (y)	Independent Variable (x)	R^2^	Regression equation	P-value
MayfarmY1	grain yield	aboveground biomass	0.95	y = 0.4251x – 152.23	<0.001
	HI	aboveground biomass	0.18	y = 0.0607ln(x) – 0.1099	0.024
		grain yield	0.44	y = 0.0772ln(x) – 0.1652	<0.001
		stover weight	0.05	y = 0.0335ln(x) + 0.123	0.292
MayfarmY2	grain yield	aboveground biomass	0.98	y = 0.6311x – 211.05	<0.001
	HI	aboveground biomass	0.84	y = 0.2003ln(x) – 0.9904	<0.001
		grain yield	0.93	y = 0.1388ln(x) – 0.4339	<0.001
		stover weight	0.63	y = 0.2596ln(x) – 1.2558	<0.001
P-farm	grain yield	aboveground biomass	0.76	y = 0.4172x + 125.76	<0.001
Westwood	grain yield	aboveground biomass	0.94	y = 0.6917x – 410.54	<0.001

### 3.2 Crop phenology model calibration

**Figure 3:**
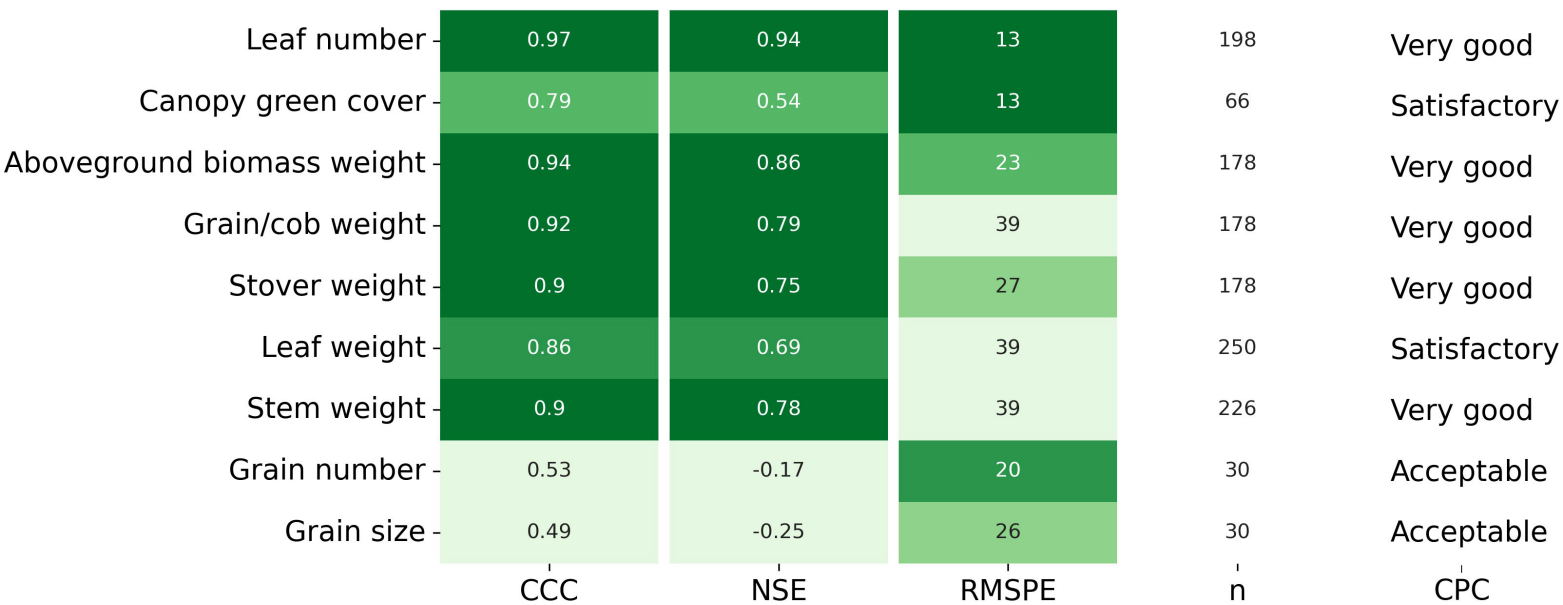
Lin’s Concordance Correlation Coefficient (CCC), Nash Sutcliffe model Efficiency coefficient (NSE), Root Mean Square Percentage Error (RMSPE), number of observations (n), and Categorical Performance Classification (CPC; Acceptable, Satisfactory, Very good) for each variable measured in-season and at final harvest. The darker green colour indicates better model performance.

**Figure 4:**
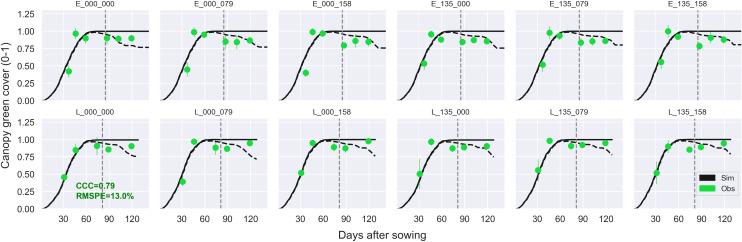
Observed (circles) and simulated (dashed black line) canopy green cover and total cover (live and dead canopy; solid black line) during the crop growing season at the MayfarmY1 experiment. Each subplot represents a treatment combination of sowing date × N fertilisation rate pre-sowing × N fertilisation rate post-sowing. For example, E_135_158 indicates an early sowing date, 135 kg N ha^-1^ is applied pre-sowing, and 158 kg N ha^-1^ is applied post-sowing. Vertical dashed lines indicate the flowering date for each treatment. The CCC and RMSPE indicate Lin’s Concordance Correlation Coefficient and the Relative Root Mean Square Percentage Error.

**Figure 5:**
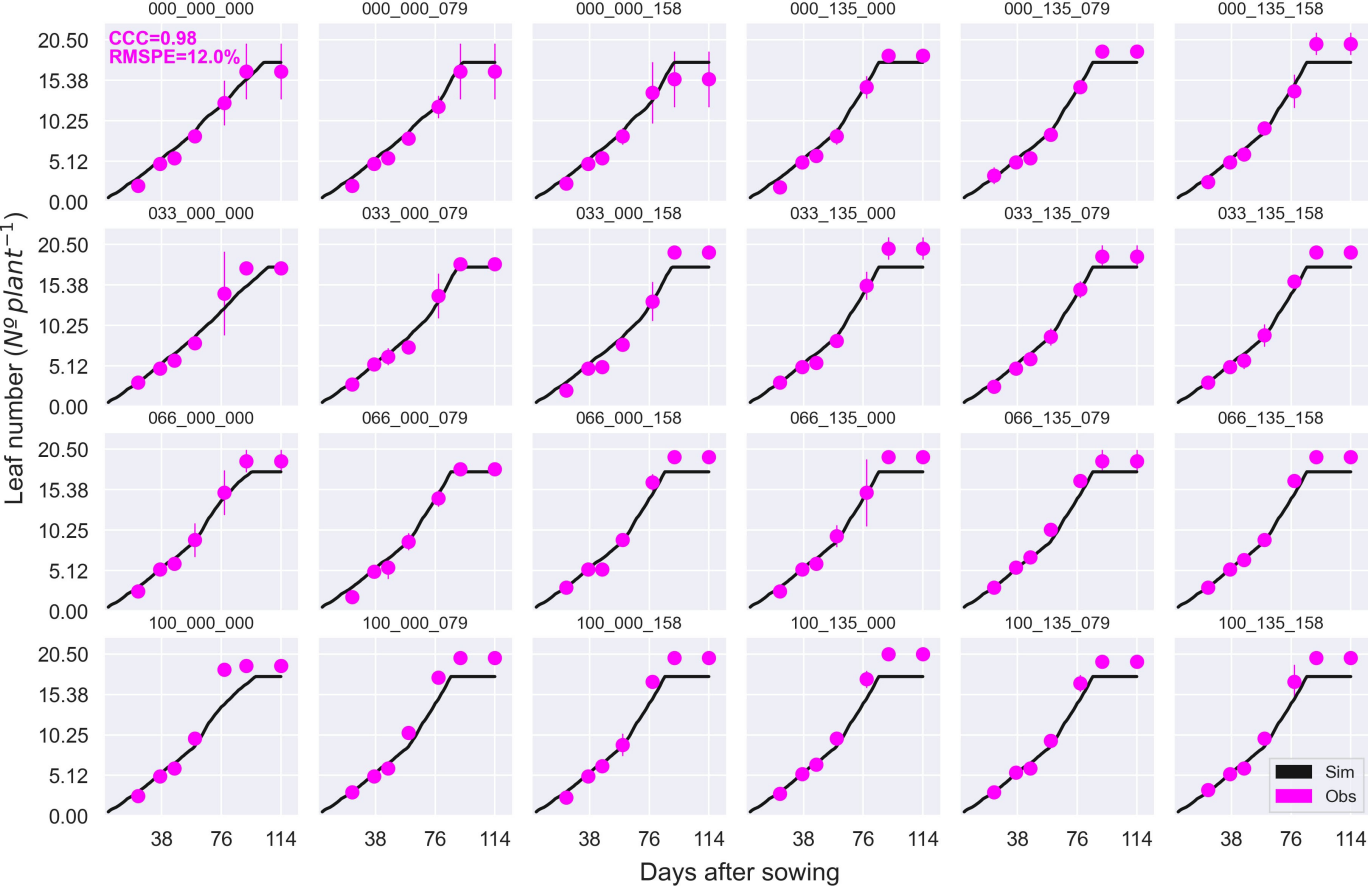
Observed (circles) and simulated (solid black line) leaf number during the crop growing season at the MayfarmY2 experiment. Each subplot represents a treatment combination of the percentage of irrigation × N fertilisation rate pre-sowing × N fertilisation rate post-sowing. For example, 100_135_158 indicates 100% irrigation, 135 kg N ha^-1^ is applied pre-sowing, and 158 kg N ha^-1^ is applied post-sowing. CCC and RMSPE indicate Lin’s Concordance Correlation Coefficient and the Relative Root Mean Square Percentage Error.

Overall, APSIM showed a very good performance to estimate crop leaf number (CCC = 0.97) and satisfactorily estimated canopy green cover (CCC = 0.79) (**Figure 3** and **Figure S2**). Therefore, the model was able to capture leaf senescence and dry matter remobilisation demonstrated by the capability to estimate green cover, which only includes active photosynthetic crop canopy (**Figure 4**). Although the ability of APSIM to estimate leaf number was very good, there were slight under-estimations of this variable under high N rates and irrigation amounts (**Figure 5**). Canopy cover variables presented the lowest RMSPE of the model calibration (13%).

### 3.3 Model calibration of crop growth and partitioning

**Figure 6:**
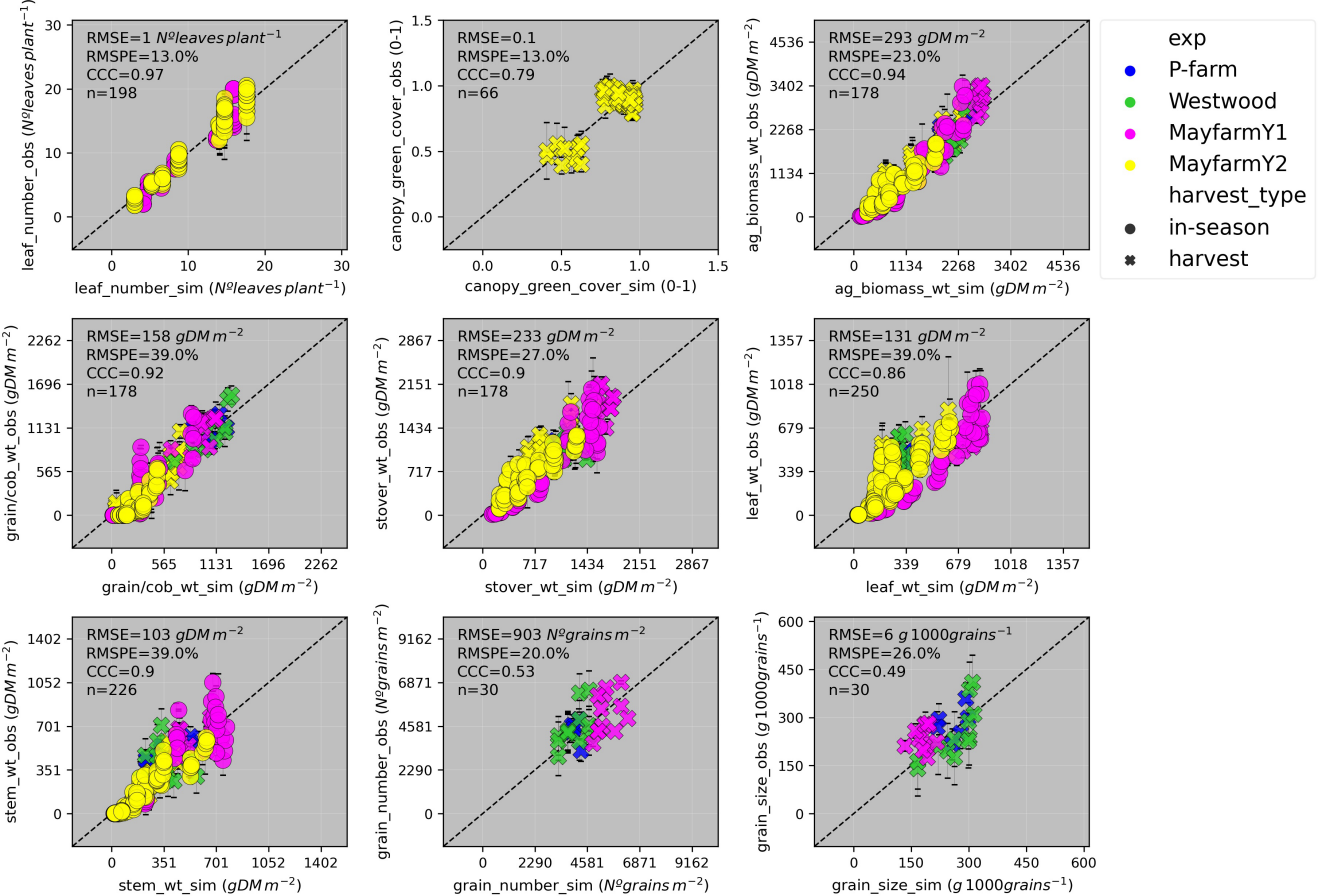
Observed vs. simulated leaf number, canopy green cover, aboveground biomass, grain + cob weight, stover weight, leaf weight, stem weight, grain number, grain size by experiment (P-farm, MayfarmY1, MayfarmY2, and Westwood), and type of harvest. Data include in-season and final harvest measurements. The solid grey line represents the line 1:1, that is, y = x and the solid black line represents the regression line adjusted to the complete dataset. RMSE, Root Mean Square Error; RMSPE, Relative Root Mean Square Percentage Error; CCC, Lin’s Concordance Correlation Coefficient; n, number of observations.

**Figure 7:**
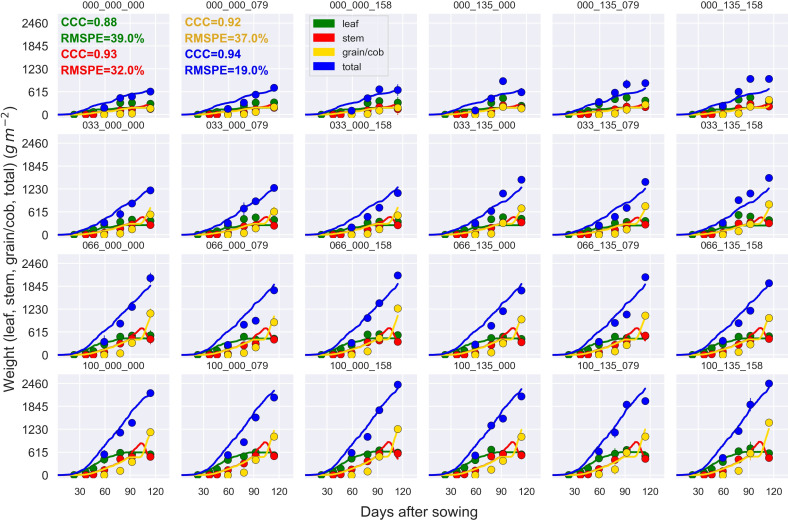
Observed (circles) and simulated (lines) leaf weight, stem weight, cob + grain weight, and aboveground biomass (total) during the crop growing season (das, days after sowing) at the MayfarmY2 experiment. Each subplot represents a treatment. For example, 100_135_158 indicates 100% irrigation, 135 kg N ha^-1^ at sowing, and 158 kg N ha^-1^ post-sowing. CCC and RMSPE indicate Lin’s Concordance Correlation Coefficient and the Relative Root Mean Square Percentage Error across treatments.

**Figure 8:**
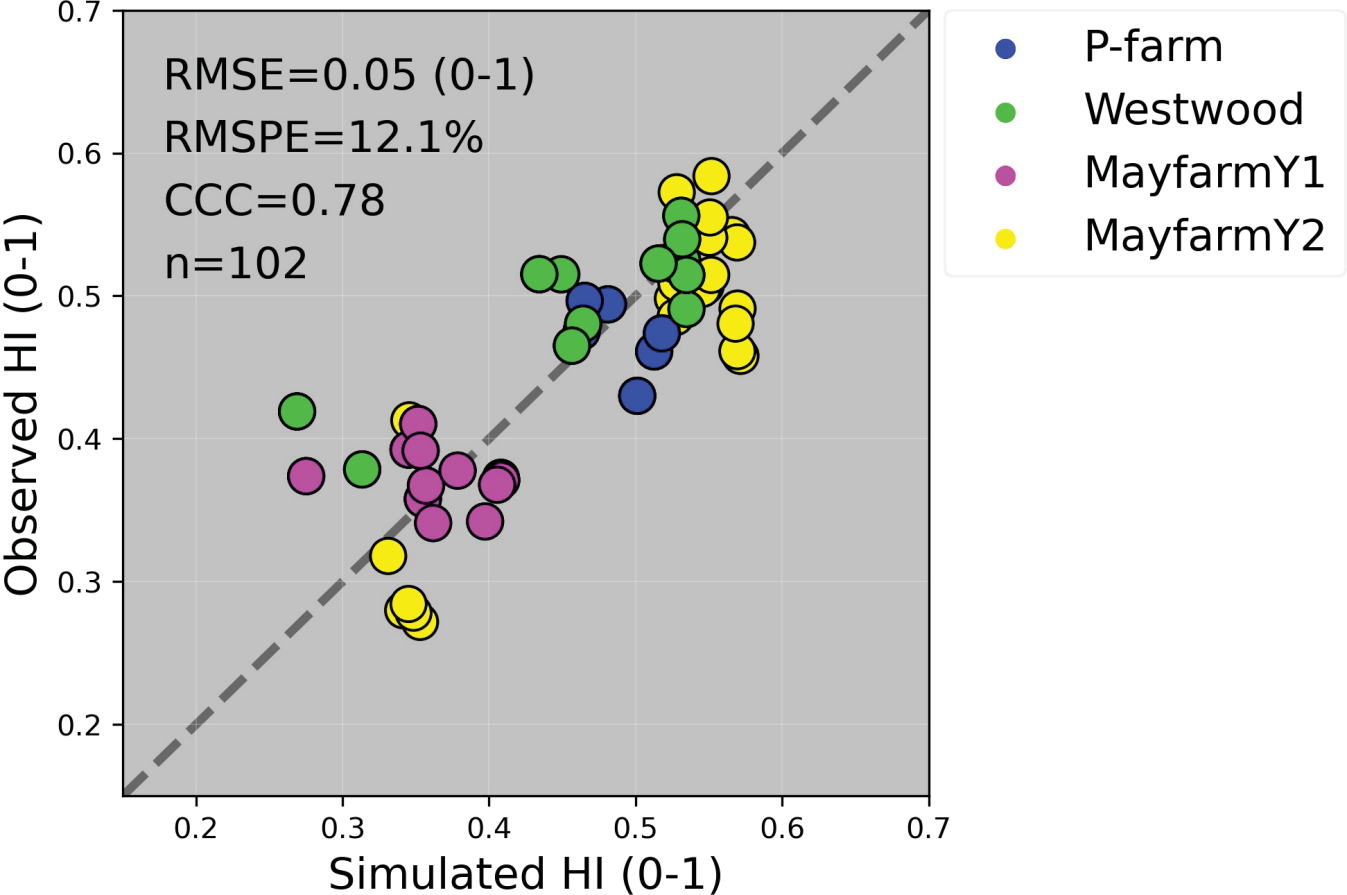
Observed vs. simulated harvest index (HI) by experiment. The solid grey line represents the line 1:1, that is, y = x and the solid black line represents the regression line adjusted to the complete dataset. RMSE, Root Mean Square Error; RMSPE, Relative Root Mean Square Percentage Error; CCC, Lin’s Concordance Correlation Coefficient; n, number of observations.

The general model assessment (i.e., using in-season and final harvest data) demonstrated that APSIM had a very good performance to simulate total aboveground biomass (CCC = 0.94), grain + cob weight (CCC = 0.92), stover weight (CCC = 0.9), and stem weight (CCC = 0.9) (**Figures 3** and **6**). Leaf weight was simulated with satisfactory model performance (CCC = 0.86). On the contrary, grain yield components (grain number and size) had an acceptable modelling performance (CCC = 0.49). The highest RMSPE was found for leaf, stem, and grain + cob weight (39%). In general, as N fertilisation (from left to right in **Figure 7** and **Figure S3**) and irrigation rates increased (from top to bottom in **Figure 7** and **Figure S3**), the model performance for different aboveground crop components increased too. Seasonal patterns of observed and simulated data demonstrated that the model was able to simulate specific crop partitioning change events. This includes the flag leaf stage, when the crop stops partitioning assimilates to the leaf component and starts translocating assimilates to the grain component (**Figure 7**). Partitioning to grain and cob started earlier in the model than in the observed data, which generated grain and cob weight over-estimations. However, the final values (close to 90 and 120 days after sowing) were satisfactorily simulated (**Figure 7**). Most importantly, the model satisfactorily estimated HI (CCC = 0.78) with a relatively low RMSPE (12.1%) (**Figure 8**). A complete statistical description of model deviations for all assessed crop variables is provided in **Table S5**.

### 3.4 Sources of model prediction uncertainty

**Figure 9:**
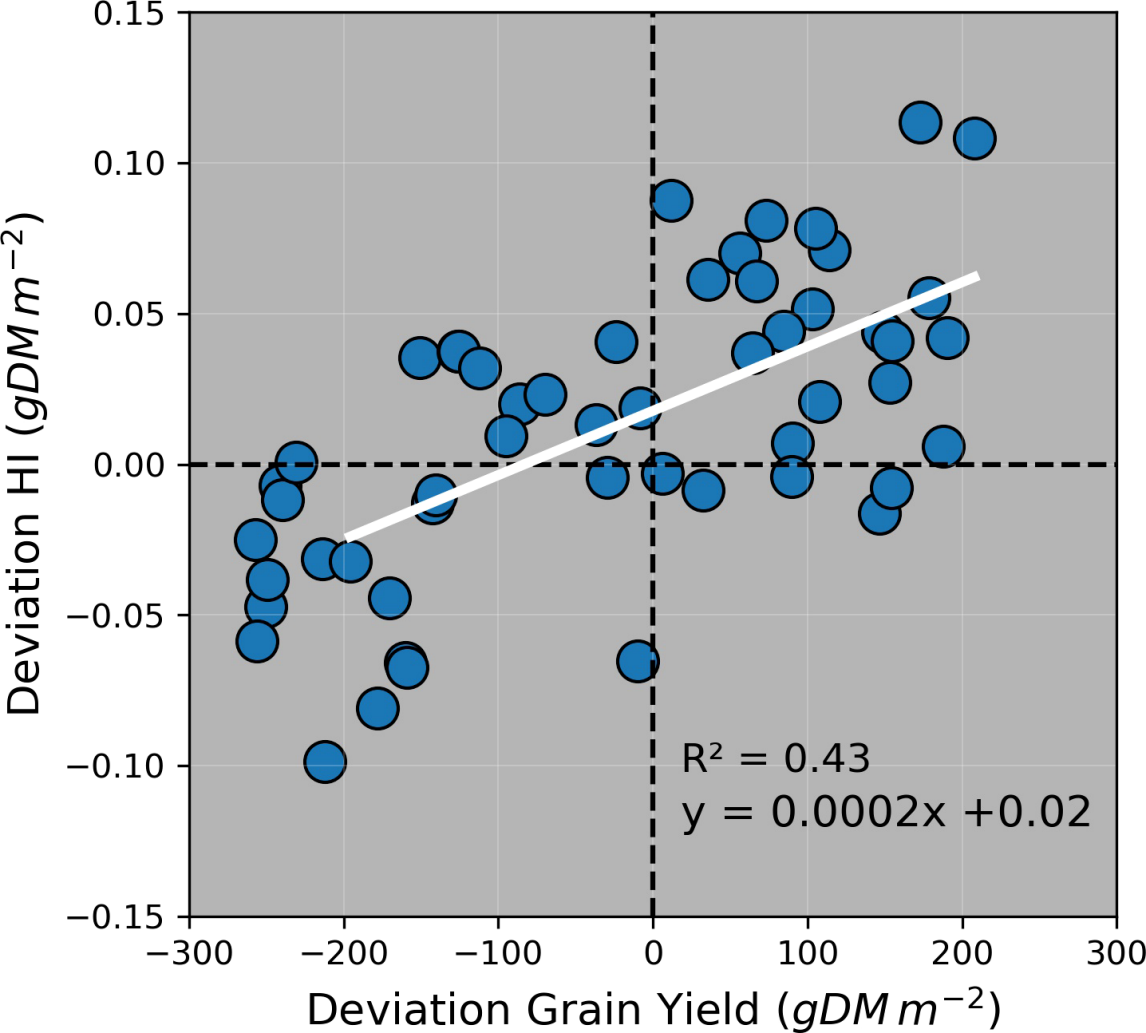
Deviation (i.e., simulated value – observed value) of harvest index (HI) vs. deviation of grain yield in all experiments. Colours and symbols indicate irrigation and N rates applied during the growing season. Dashed horizontal and vertical lines represent no deviation between simulated and observed values.

The ability of the model to estimate the maize HI was mainly affected by the accuracy to estimate grain yield (**Figure 9**). Although the model prediction uncertainty (simulated values – observed values) was higher than the observed standard deviations for most crop variables, it was equal and lower than the observed standard deviations for grain number and grain size, respectively (**Figure S4**). This demonstrated that the model was able to capture the variability of biomass partitioning across treatments and environments. The model prediction uncertainty was also affected by the sampling method applied for specific crop variables (leaf weight, stem weight, grain yield + cob weight, and aboveground biomass), particularly for grain yield + cob weight (**Figure S5**).

### 3.5 Assessing the harvest index variability through scenario analysis

**Figure 10:**
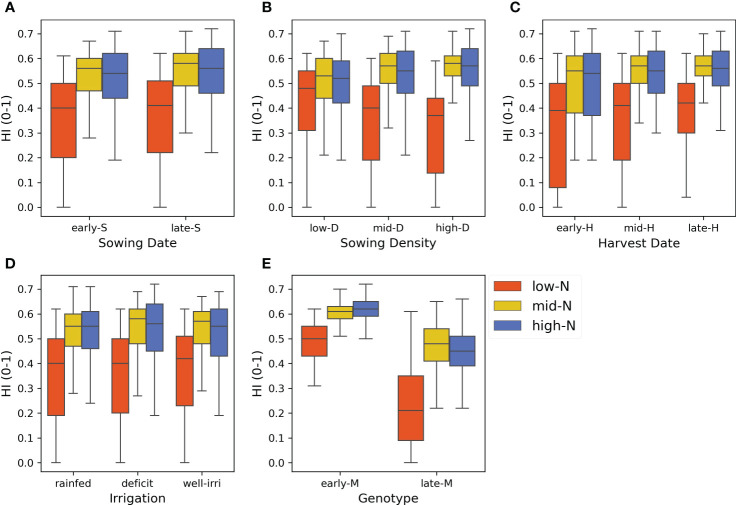
Harvest index (HI) variance for each level of crop management factor (subplots). **(A)** Sowing dates (20 Oct and 3 Nov), **(B)** sowing density (5.2, 7.2, and 9.3 pl m^-2^), **(C)** harvest date (125, 138, and 150 days after sowing), **(D)** irrigation (rainfed, deficit, and well-irrigated), **(E)** genotype (early and late maturity) across N application rates in colours (0, 125 and 250 kg N ha^-1^), and years (50 seasons).

**Figure 11:**
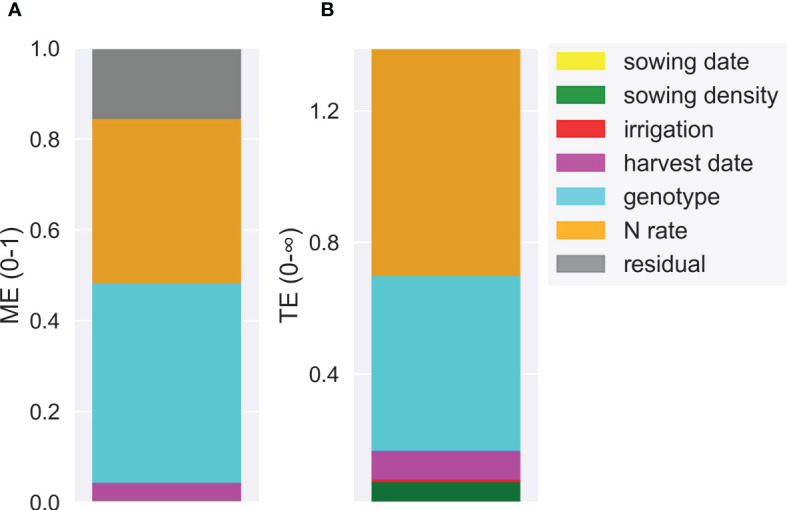
Main effect (ME) **(A)** and total effect (TE) **(B)** of different crop management factors (sowing date, sowing density, harvest date, irrigation, genotype, and N rate) explaining simulated harvest index variance.

The scenario analysis demonstrated that genotype and N application rates were the most influential crop management factors affecting the variance of HI (**Figures 10** and **11**). Nitrogen rate outweighed the effect of irrigation which strongly affected grain yield, and therefore HI, independent of the water status (**Figures S6** and **S7**). The harvest index varied from 0 (for scenarios without N rate applications) to 0.71 (for late sowing × high density × early genotype scenarios) (**Table S6**). The median HI across years and factors was 0.51. Genotype and N rate contributed to 44% and 36% of the HI variance (ME = 0.44 and ME = 0.36, respectively) (**Figure 11A**). These factors were also the most interactive across all crop management practices (**Figure 11B**). Harvest date, sowing density, sowing date, and irrigation had a lower contribution to HI variance (ME< 0.04).

## 4 Discussion

### 4.1 Drivers of observed grain yield and harvest index variability

We showed how genotype impacted the partitioning of maize assimilate to cob and HI and the ratio between HI and stover proportion (HI : SP) across experiments (**Figure 2**) , but the main driver of observed HI variability for a given genotype was crop water status (**Figure S1**). These results align with previous studies which assessed the effect of maize genotypes under a wide range of water conditions (Gambin et al., 2016). Harvest index has been shown to be affected by various biotic and abiotic stresses, including water, temperature, N, diseases, and pests (Bender et al., 2013; Khalili et al., 2013; Hütsch and Schubert, 2017; Liu et al., 2022). Our results highlight the importance of genotype selection (based on its HI : SP) and the maintenance of optimum water conditions during the maize growing season in order to achieve high HI and, therefore, greater silage quality (Hernandez et al., 2020).

Interestingly, the crop growing period driven by the thermal time between sowing and flowering and between flowering and maturity was similar between the genotypes used in this work (**Table 3**). In fact, although the HI in MayfarmY1 was significantly lower than in the other experiments, the genotype used in this experiment had a similar vegetative:reproductive thermal time ratio to other genotypes, such as PioneerX in Westwood (**Table 3**). For a given genotype, increasing grain number per plant or decreasing vegetative shoot biomass are the two main manipulations for increasing maize HI (Hütsch and Schubert, 2017). As such, the ideal maize plant for silage would have both high vegetative shoot biomass and a high grain number per plant (Johnson et al., 1999). Although the duration of the vegetative and reproductive phases may change HI (Capristo et al., 2007), the growing conditions explored during the critical period for grain set and the grain filling period and the intrinsic grain set efficiency of a given genotype are the main factors governing this variable in maize (Tollenaar et al., 2006). Accordingly, modern maize hybrids have similar vegetative biomass at flowering but a lower shoot-dry matter threshold for yield when compared with older maize hybrids, mainly due to greater biomass partitioning to cobs, higher kernel set efficiency, and more grain number per plant (Ciancio et al., 2016). At the same time, a lower susceptibility of the crop to low soil water availability could be the cause of greater grain yield stability (Messina et al., 2022). In this study, we found maximum grain yields (1,159 g m^-2^; **Figure 2B**) at MayfarmY1 under well-irrigated treatments (accumulated irrigation during growing season = 534 mm), but the HI in this experiment was on average the lowest (0.37; **Figure 2D**). One reason for this may be the genotype used in this experiment which produced a greater stover proportion with a reduced partitioning to cobs (**Figure S1C**). Variability in HI has been observed between silage maize genotypes (Tsakmakis et al., 2021), supporting the genotypic patterns observed in the present work. Our results highlight the need to incorporate a wide range of genotype maturity types and the HI : SP ratio in further analyses when HI of silage genotypes are compared across environments.

### 4.2 Drivers of simulated grain yield and harvest index variability

We calibrated APSIM to estimate crop growth, development, and plant biomass partitioning (and therefore HI) and explored the main sources of prediction uncertainty. Four detailed crop experiments on maize grown for silage were used to successfully calibrate APSIM, enhancing the value and applicability of historical field experimental data not collected for crop modelling purposes. Although we calibrated APSIM for biomass partitioning and grain yield, we carried it out on a more detailed level than common calibration practices usually performed in published papers, including variables such as grain size and grain number which usually are not included. This paper calibrated APSIM for forage maize using 1,322 field observations across a wide range of factors (sowing date, sowing density, genotype, N rate, and irrigation), highlighting the novelty and uniqueness of this calibration. Also, we calibrated the model in parallel for nine model outputs, i.e., we considered the trade-offs between state variables in the model using this approach.

Previous studies parametrised APSIM using datasets from Australian environments and grain maize genotypes (Hammer et al., 2010; Soufizadeh et al., 2018; Rotili et al., 2020). However, in this study, we calibrated this model for maize growth in Australia across nine crop variables, five silage genotypes, and six factors of analysis (**Table 1**). Although APSIM has been used to simulate the biomass production of silage maize under temperate environments (Pembleton et al., 2013; Teixeira et al., 2017; Ojeda et al., 2018a; Ojeda et al., 2018b) and extremely high latitudes (Morel et al., 2020), the model has been not previously tested for its capacity to predict the biomass partitioning for silage genotypes. Our work is the first study that provides reasonable APSIM estimations of grain yield and aboveground biomass (**Figures 3** and **6**) and satisfactory estimations of HI (**Figure 8**). The estimations of grain number and grain size presented in this study are comparable with other results reported in Gatton, Australia, (Rotili et al., 2020) although the prediction uncertainty for grain size in our work was considerably lower (RMSE = 6 mg grain^-1^ vs. 38 mg grain^-1^). These results provide confidence to use the model for nutritive quality predictions for maize silage as this crop’s HI is correlated with neutral detergent fibre content and *in vitro* true digestibility (due to different grain:stover ratio through a HI gradient) (Tsakmakis et al., 2021). Overall, the model overpredicted HI when the crop was growing underwater limitations (**Figure 9**); however, these overestimations were relatively low on average (HI = 0.04). Therefore, this paper demonstrates that APSIM can model a set of crop traits and processes that directly affect the silage quality, expecting deviations lower than 10% in HI predictions (considering the average observed HI was 0.465).

In this study, we also demonstrated that a satisfactory model calibration could be achieved by parametrising the crop model for both phenology and crop growth. It is particularly important to properly simulate the occurrence of key crop stages related to carbon allocation to different plant components (Brown et al., 2019) and, therefore, define the HI and stover proportion derived from leaf and stem partitioning. Previous modelling studies have applied several methods to create new genotypes in crop models, including the minimisation of statistical metrics (e.g., RMSE) between observed and simulated values (Messina et al., 2006), gene-to-phenotype multi-trait link function integration (Cooper et al., 2021), and sensitivity analysis of cultivar trait parameters (Sexton et al., 2017). However, in this research, we conducted an exhaustive and robust procedure to generate new silage genotypes in the model, integrating model parametrisation (using observations to reduce the RMSE) and model optimisation based on sensitivity analysis (Monod et al., 2006) (**Figure 1**).

Previous studies showed the strong effect of genotype and N application rates on maize grain yield (Rossini et al., 2016; Ruiz et al., 2022). These highlighted the importance of the maize growing period length (particularly the reproductive phase) and crop N availability to determine HI. However, some specific features of our study are novel when compared to the previous knowledge. Genotype and N application rate were the main drivers of simulated HI variability across years (i.e., under contrasting rainfall patterns) (**Figure 10E**), which corresponded with our findings from field experimental data (Section 4.1; **Figure 2**). However, the differences in HI were higher between genotypes under N-limited crops (N rate = 0 kg N ha^-1^) than N fertilised crops (125 and 250 kg N ha^-1^) because late maturity genotypes had higher biomass in relation to grain yield than early maturity genotypes, which considerably reduced HI (**Figure 10E**). This highlights the importance of N fertilisation to achieve high HI, independent of the genotype used.

The in-silico simulations allowed us to capture the interactions between N rate application and other factors of variance (mainly irrigation) across years, something that was not possible to identify in the field despite using a similar range of N application rates (0-158 kg N ha^-1^) due to lack of inter-annual rainfall variability. Maize biomass and grain yield maximisation are usually conditioned by the existence of water and N co-limitation, in which limitations due to one resource depend on limitations due to the other resource (Cossani and Sadras, 2018). The response to the addition of one resource depends on the level at which the other is limiting (obeying the law of optimum rather than the law of minimum). The ability of the crop to uptake N depends on the water stress (Hammad et al., 2017); water-stressed crops have high N concentrate, and synergistic effects have been found between N and water stress in maize (Rossini et al., 2016). In our simulated scenarios, the responses of HI to irrigation were only expressed under non-N limited conditions (>125 kg N ha^-1^), indicating that the effect of N rate on grain yield and, therefore HI, outweighed the effect of irrigation. It must be noted that the synergistic effects of water and N stress depend on the magnitude and timing of both stresses (Rossini et al., 2016), and the constant magnitude of the water limitations simulated across the whole crop cycle could have conditioned the co-limitation level. Also, the long-term analysis showed that maize crops limited by N (N rate = 0 kg N ha^-1^) had much lower vegetative biomass than under high-N rates and, therefore, under these conditions, the crop generally did not experience water stress due to the amount of soil water available, which was enough to provide for the low water requirements under those conditions (**Figure S6**). The main differences of HI were found between the treatment with 0 *vs.* 125 kg N ha^-1^ of applied N, while HI was maximised above 125 kg N ha^-1^ and therefore, the results of this scenario analysis suggest that, under high-rainfall years, the treatment with 125 kg N ha^-1^ maximises HI and, therefore, can potentially improve maize silage quality.

### 4.3 Implications of this study and future work

This work provides a solid basis for additional parameterisation of APSIM for silage maize with the overarching goal of accurately predicting changes in plant’s partitioning and composition and, from these, potentially predicting the nutritive value of whole plant maize silage. As previously highlighted by Archontoulis et al. (2014), accurate calibration of crop phenology is the primary priority as the partitioning of carbohydrates strongly depends on the phenology stage. Our results showed the need to integrate different approaches to achieve accurate genotype parameters to reproduce the G×M×E interactions in the model. This was demonstrated by the high accuracy to estimate grain yield independent of the genotype, environment, and crop management conditions (**Figure 6**). Therefore, future studies should focus on a detailed phenological parametrisation of the genotypes used to calibrate models using field or remote sensing-based phenological data as demonstrated in this study and by Yang et al. (2022) and Della Nave et al. (2022).

In addition, the overall satisfactory performance of APSIM to accurately predict phenology and HI found in this study has several implications for future work. A number of silage quality components are directly correlated with HI (i.e., starch content, neutral detergent fibre content, and forage digestibility) (Tsakmakis et al., 2021; Jahangirlou et al., 2023). Therefore, further work should be conducted to calibrate the model to estimate these quality components and quantify the prediction uncertainty. Further work is also required to determine the impact of N plant content on silage crude protein, starch, fibre, and energy and their interactions with HI to allow the modelling of these fine-tuning relationships from a functional perspective. Moreover, these results may be used to estimate the grain proportion of silage in advance (i.e., from the early stages of the crop), guiding diet balance and supplement purchases at the farm level. Future work should be done by integrating this kind of analysis across regions and at the farm level which will allow to cover a broader range of soil types and spatial soil variability.

We found modelling performance to differ when the same variables were collected using different harvest methodologies, i.e., harvesting the whole plot vs. proportions determined from one sampled plant per plot (**Figure S5**). This is particularly important because it generates uncertainty in the observations, which generally is not accounted for in crop modelling studies as a source of uncertainty (Chapagain et al., 2022). The quantification of the observational uncertainty is a key step in any model calibration procedure as it allows model users to target different model accuracy thresholds to finish the calibration. It highlights the need to use common protocols for crop sampling if experiments are targeted for crop modelling purposes, particularly for biomass partitioning. Future modelling studies should carefully consider the ground sampling method applied to calibrate models for crop biomass partitioning.

## 5 Conclusions

In this study, we present the first crop modelling study that calibrates APSIM for HI and plant partitioning using a detailed field dataset of forage maize and considering nine crop variables simultaneously. While field experimentation during one growing season demonstrated that the main drivers of HI variability were genotype and water status, the long-term scenario analysis highlighted the importance of N rate application as a second driver. We also applied an integrated and robust approach for phenology and crop growth parameter calibration. In this study, we demonstrated that the calibrated APSIM model:

- has a satisfactory performance using five silage genotypes for maize grown in Australia across a wide range of G×E×M combinations. This is one of the first attempts to use a crop model to predict silage maize HI under contrasting crop growth scenarios.
- is a suitable tool to estimate maize HI as a potential proxy of silage quality for forage purposes.
- could now be used to compare inter-annual variability of maize HI based on G×E×M interactions and, therefore, assist in real-world farming conditions towards better synchronisation of crop management interactions focusing on high maize silage quality.

## Data availability statement

The raw data supporting the conclusions of this article will be made available by the authors, without undue reservation.

## Author contributions

JO, RI, and SG contributed to the conception and design of the study. JO and RI organised the database. JO performed the statistical analysis and modelling. JO wrote the first draft of the manuscript. All authors contributed to the article and approved the submitted version.
