# Supplementary material for: Field and in-silico analysis of harvest index variability in maize silage
Source: Front Plant Sci. 2023 Jun 19;14:1206535. doi: 10.3389/fpls.2023.1206535 (PMC10316513; doi:10.3389/fpls.2023.1206535)
Supplement: Supplementary file 1 [file DataSheet_1.docx]

Supplementary Material

**Field and in-silico analysis of harvest index variability in maize silage**

**Jonathan Jesus Ojeda^*^, Md Rafiq Islam,** **Martin Correa-Luna, Juan Ignacio Gargiulo, Cameron Edward Fisher Clark, Diego Hernán Rotili, Sergio Carlos Garcia**

*** Correspondence**

Jonathan Jesus Ojeda

[jonathanjesusojeda@gmail.com](mailto:jonathanjesusojeda@gmail.com)

# 1. Supplementary Figures


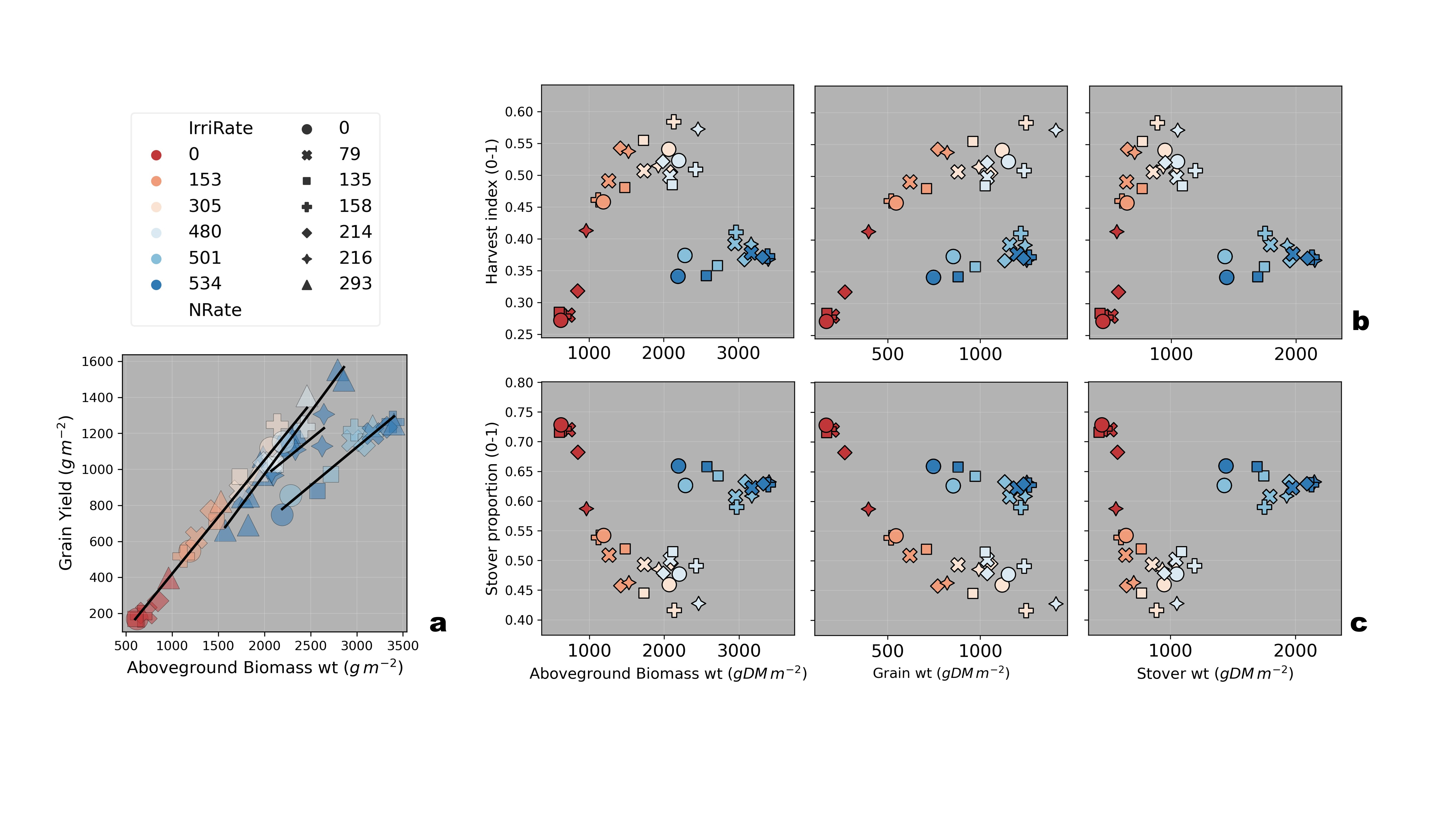


**Supplementary Figure 1.** (a) Grain yield vs aboveground biomass, (b) observed harvest index (grain yield/aboveground biomass) and (c) stover proportion (stover weight/aboveground biomass) vs. observed aboveground biomass, stover and grain weight at final harvest by irrigation rate (IrriRate; mm) and N rate (NRate; kg N ha^-1^). Panels b and c show data for MayfarmY1 and MayfarmY2 experiments.


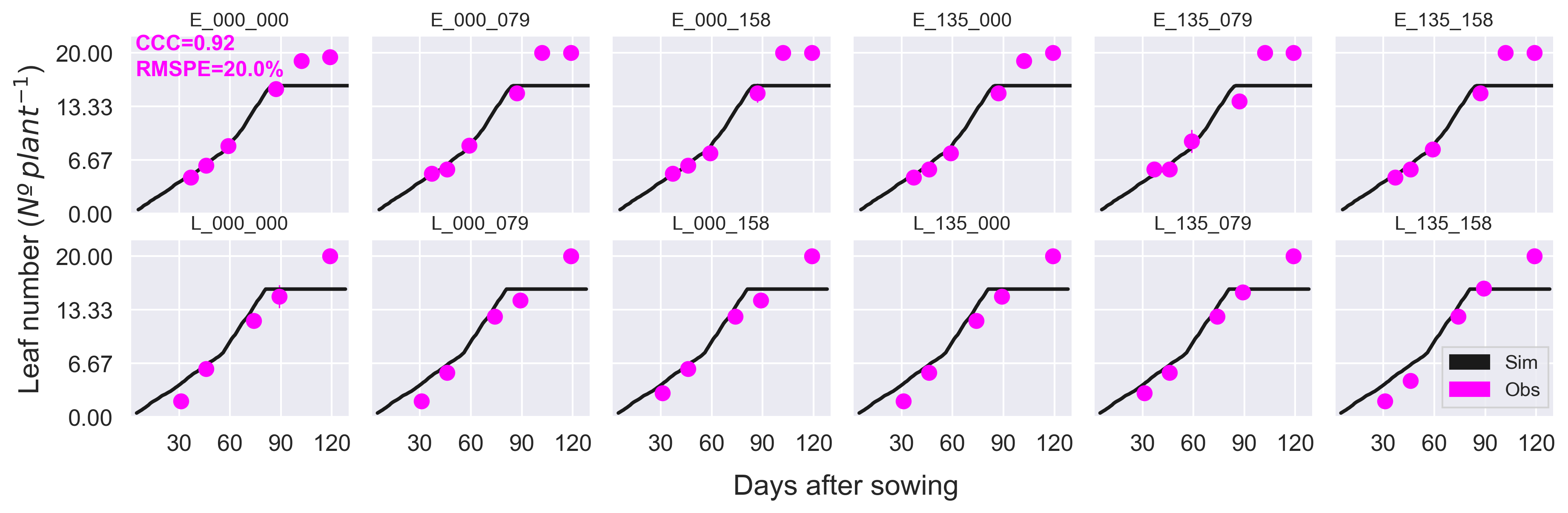


**Supplementary Figure 2.** Observed (circles) and simulated (solid black line) leaf number during the crop growing season (das, days after sowing) at the MayfarmY1 experiment. Each subplot represents a treatment. For example, E_000_000 indicates early sowing, 0 kgN ha^-1^ at sowing and 0 kgN ha^-1^ post-sowing. CCC and rRMSE indicates the Lin's Concordance Correlation Coefficient and the Relative Root Mean Square Percentage Error across treatments.


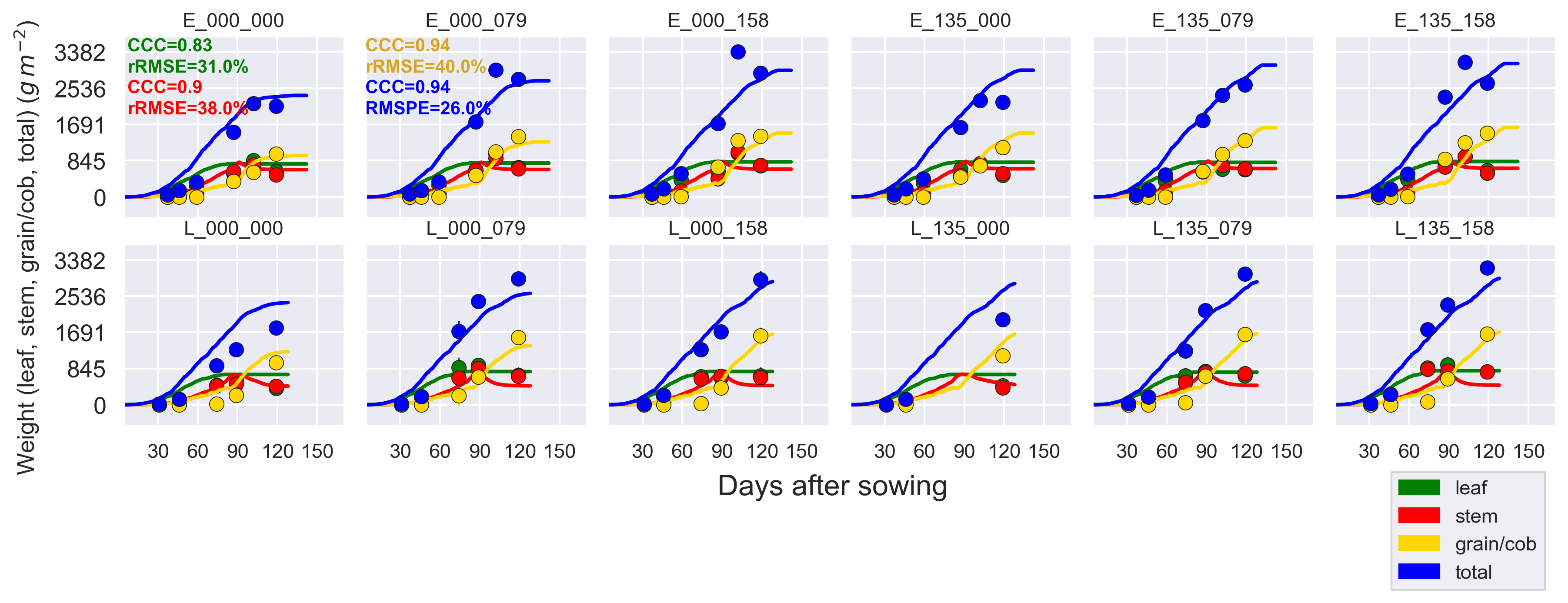


**Supplementary Figure 3.** Observed (circles) and simulated (lines) leaf weight, stem weight, cob + grain weight and aboveground biomass (total) during the crop growing season (das, days after sowing) at the MayfarmY1 experiment. Each subplot represents a treatment. For example, E_000_000 indicates early sowing, 0 kg N ha^-1^ at sowing and 0 kg N ha^-1^ post-sowing. CCC and rRMSE indicates the Lin's Concordance Correlation Coefficient and the Relative Root Mean Square Percentage Error across treatments.


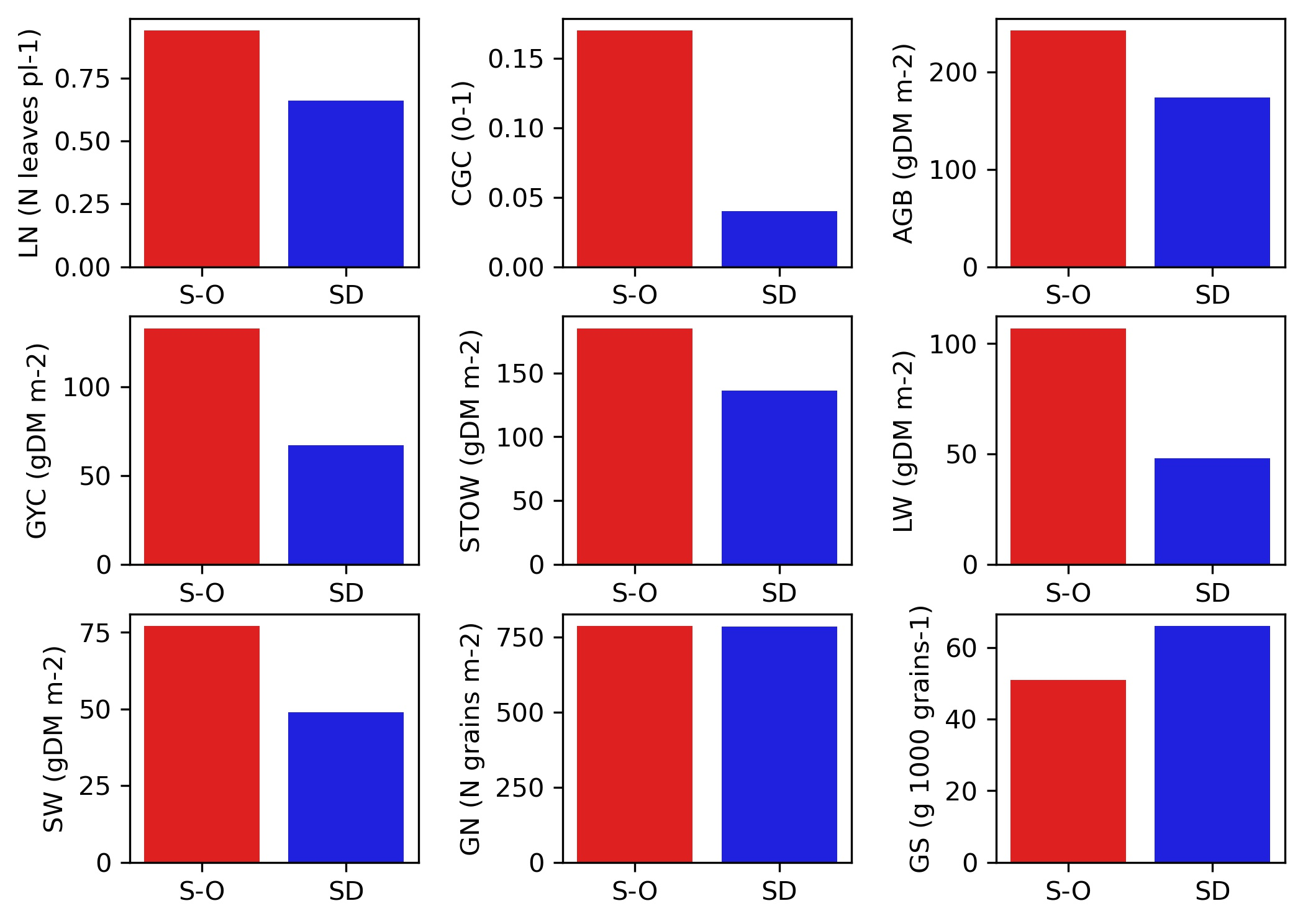


**Supplementary Figure 4.** Mean prediction error (S-O) and observed standard deviation (SD) by variable assessed in the model calibration. LN, leaf number; CGC, canopy green cover; AGB, aboveground biomass; GYC, grain yield + cob weight; STOW, stover weight; LW, leaf weight; SW, stem weight; GN, grain number; GS, grain size.


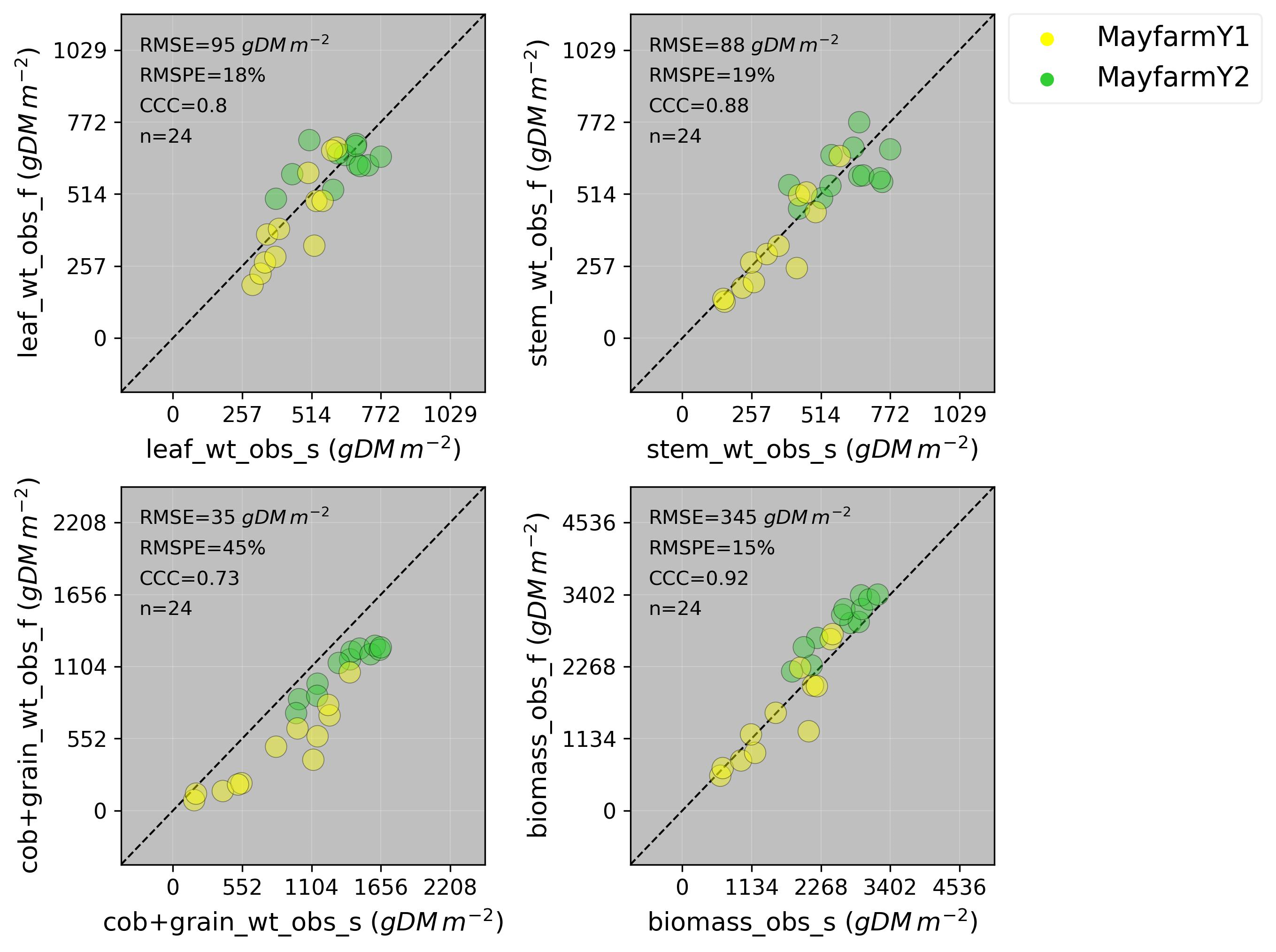


**Supplementary Figure 5.** Observed leaf weight, stem weight, cob + grain weight and aboveground biomass using a plot sampling method (y-axis) vs a plant sampling method (x-axis) in the MayfarmY1 and MayfarmY2 experiments. The solid grey line represents the line 1:1, that is, y = x and the solid black line the regression line adjusted to the complete dataset. RMSE, Root Mean Square Error; RMSPE, Relative Root Mean Square Percentage Error; CCC, Lin's Concordance Correlation Coefficient; n, number of observations.


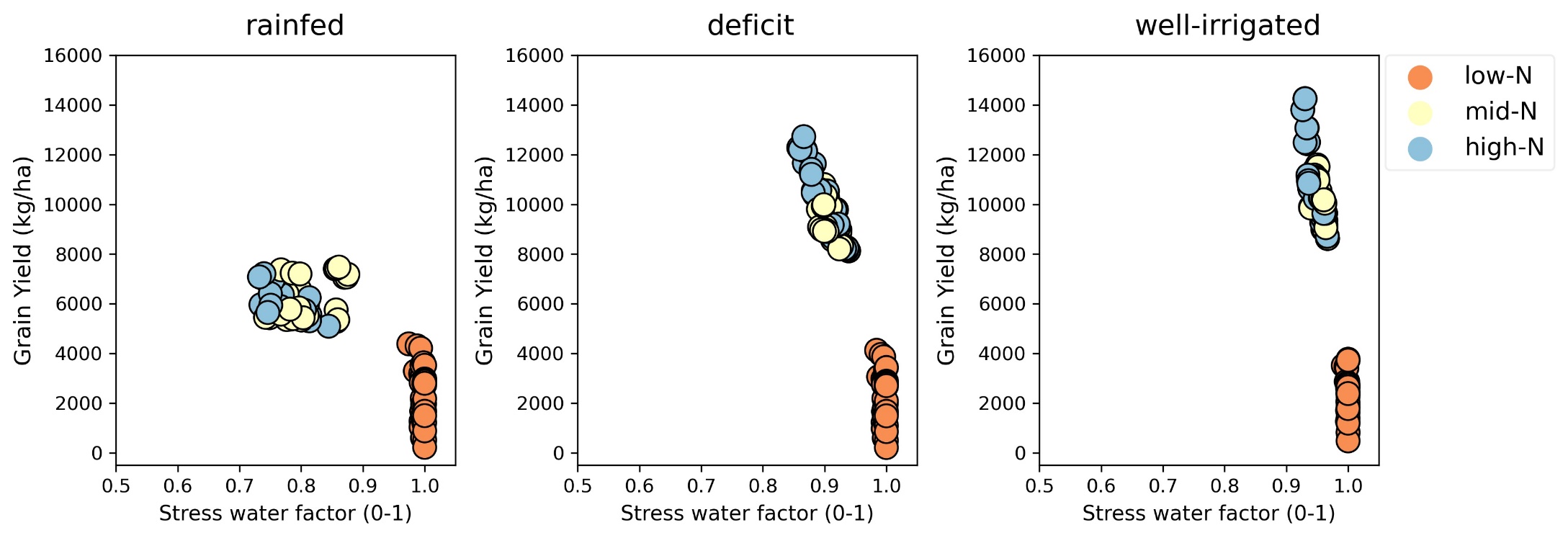


**Supplementary Figure 6.** Simulated grain yield vs mean water stress deficit across the growing season with lower mean rainfall (160 mm) by irrigation treatment (rainfed, deficit and well-irrigated) and N rate application (low-, mid- and high-N). A water stress factor of 0 is complete stress and 1 no stress.


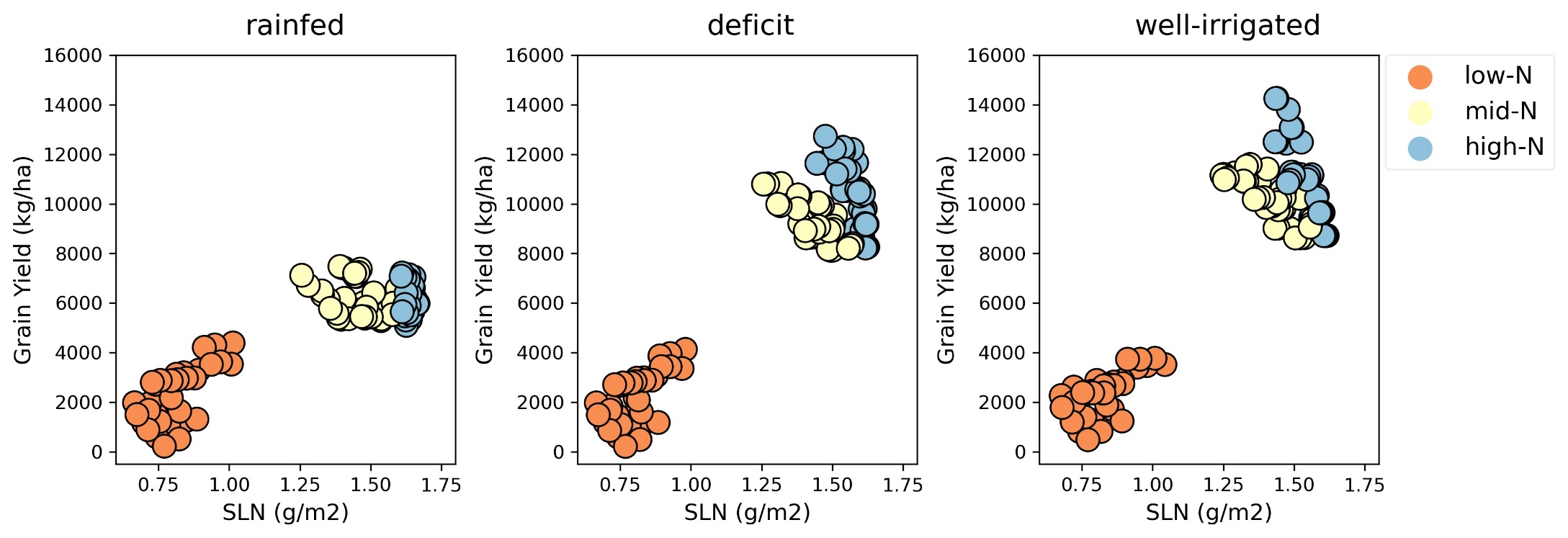


**Supplementary Figure 7.** Simulated grain yield vs mean Specific Leaf Nitrogen (SLN) across the growing season with lower mean rainfall (160 mm) by irrigation treatment (rainfed, deficit and well-irrigated) and N rate application (low-, mid- and high-N). Lower and higher SLN indicate complete stress and no stress, respectively.

**2. Supplementary Tables**

**Supplementary Table 1.** Description of the most important model processes that affects biomass and crop yield estimations in the APSIM-Maize model.

| **Process** | **Description** |
| --- | --- |
| *Temperature* | There are 11 crop stages and nine phases (time between stages), and commencement of each stage (except for sowing to germination which is driven by soil moisture) is determined by accumulation of thermal time. Photosynthesis is also affected by temperature through a stress function applied to the daily radiation use efficiency (RUE). RUE is optimal between 15 and 35 °C. |
| *RUE* | RUE is a constant value (1.85 g MJ^-1^) and this model parameter is multiplied by adjustment factors to account for the effects of water, temperature, N, vapour pressure deficit and atmospheric carbon dioxide concentration stresses (Ojeda et al., 2020). |
| *Transpiration* | Transpiration efficiency is derived from a transpiration efficiency coefficient (0.009 kPa) and the vapour pressure deficit estimated from daily temperatures. |
| *Evapotranspiration* | FAO‐56 Penman‐ Monteith (H. Brown et al., 2014) adapted by Snow and Huth (2004). |
| *Water and N stress* | Three water deficit factors are calculated which correspond to four plant processes each having different sensitivity to water stress i.e., photosynthesis, phenology, and leaf expansion. A water availability ratio is calculated by dividing actual soil water supply by the potential soil water supply (DUL). This ratio is used in the relationships illustrated to derive the water stress factors for photosynthesis and leaf expansion.  Daily thermal time values are cumulated into a thermal time sum which is used to determine the duration of each phase. Between the stage of emergence and flowering the cumulated daily thermal time is reduced by water or N stresses, resulting in delayed phenology when the crop is under stress. |

**Supplementary Table 2.** Soil parameter description used to run APSIM for all experiments.

| **Depth** | **BD** | **AirDry** | **LL15** | **DUL** | **SAT** | **OC** | **pH** | **N-NO_3_** | **N-NH_4_** | **Initial water** | **Maize LL15** | **PAWC** | **KL** | **XF** |
| --- | --- | --- | --- | --- | --- | --- | --- | --- | --- | --- | --- | --- | --- | --- |
| *cm* | *g cm^-3^* | *mm mm^-1^* | | | | *%* | *1:5 water* | *ppm* | *ppm* | *%* |  | *mm* | *mm d^-1^* | 0-1 |
| 0-15 | 1.343 | 0.054 | 0.16 | 0.31 | 0.43 | 2.27 | 5.552 | 16.2 | 3 | 100 | 0.172 | 20.1 | 0.06 | 1 |
| 15-30 | 1.463 | 0.055 | 0.16 | 0.29 | 0.4 | 1.24 | 5.615 |  |  | 100 | 0.172 | 17.1 | 0.06 | 1 |
| 30-45 | 1.473 | 0.059 | 0.18 | 0.29 | 0.4 | 0.81 | 5.644 |  |  | 100 | 0.183 | 16.7 | 0.06 | 1 |
| 45-60 | 1.454 | 0.062 | 0.19 | 0.3 | 0.41 | 0.65 | 5.671 |  |  | 100 | 0.191 | 16.9 | 0.06 | 1 |
| 60-80 | 1.471 | 0.063 | 0.19 | 0.31 | 0.4 | 0.53 | 5.74 |  |  | 100 | 0.194 | 22.2 | 0.05 | 1 |
| 80-100 | 1.5 | 0.063 | 0.19 | 0.3 | 0.39 | 0.45 | 5.815 |  |  | 100 | 0.194 | 21.6 | 0.04 | 1 |
| 100-120 | 1.5 | 0.063 | 0.19 | 0.3 | 0.39 | 0.45 | 5.815 |  |  | 100 | 0.194 | 21.6 | 0.04 | 1 |
| 120-200 | 1.5 | 0.063 | 0.19 | 0.3 | 0.39 | 0.45 | 5.815 |  |  | 100 | 0.194 | 86.4 | 0.04 | 1 |

BD = bulk density; LL15 = soil lower limit or wilting point; DUL = drained upper limit or field capacity; SAT = saturated volumetric water content; OC = organic carbon; pH = pH in a 1:5 suspension of soil in water; N-NO_3_, Nitrogen from soil nitrates; N-NH_4_, nitrogen from soil ammonia; Maize LL15, crop lower limit or wilting point; PAWC = plant available water capacity per soil layer; KL, root water extraction value; XF, root exploration factor.

**Supplementary Table 3.** Description of crop management used to parametrise APSIM.

| **Experiment** | **Genotype** | **Sowing date** | **Harvest date** | **Sow**  **density** | **Dry**  **matter** | **N fert rate 1** | **N fert rate 2** | **Total N rate** | **N fert date 1** | **N fert date 2** | **Irrigation**  **amount** |
| --- | --- | --- | --- | --- | --- | --- | --- | --- | --- | --- | --- |
|  |  |  |  | ***pl m^-2^*** | ***%*** | ***kgN ha^-1^*** | ***kgN ha^-1^*** | ***kgN ha^-1^*** |  |  | ***mm*** |
| MayfarmY1 | PioneerY | 20/10/2008 | 16/02/2009 | 8.4 | 30.8 | 0 | 0 | 0 | 20/10/2008 | 04/12/2008 | 501 |
|  |  |  |  | 9.1 | 29.8 |  | 79 | 79 |  |  |  |
|  |  |  |  | 10.0 | 28.5 |  | 158 | 158 |  |  |  |
|  |  |  |  | 9.5 | 30.4 | 135 | 0 | 135 |  |  |  |
|  |  |  |  | 9.3 | 29.6 |  | 79 | 214 |  |  |  |
|  |  |  |  | 10.0 | 29.0 |  | 158 | 293 |  |  |  |
|  |  | 03/11/2008 | 02/03/2009 | 7.6 | 29.1 | 0 | 0 | 0 | 03/11/2008 | 18/12/2008 | 534 |
|  |  |  |  | 10.6 | 30.0 |  | 79 | 79 |  |  |  |
|  |  |  |  | 10.5 | 29.4 |  | 158 | 158 |  |  |  |
|  |  |  |  | 7.5 | 29.6 | 135 | 0 | 135 |  |  |  |
|  |  |  |  | 9.5 | 28.3 |  | 79 | 214 |  |  |  |
|  |  |  |  | 11.4 | 29.5 |  | 158 | 293 |  |  |  |
| MayfarmY2 | Pioneer 31H50 | 20/10/2009 | 12/02/2010 | 10 | 27.3 | 0 | 0 | 0 | 20/10/2009 | 04/12/2009 | 0 |
|  |  |  |  |  | 21.0 |  | 79 | 79 |  |  |  |
|  |  |  |  |  | 23.4 |  | 158 | 158 |  |  |  |
|  |  |  |  |  | 23.0 | 135 | 0 | 135 |  |  |  |
|  |  |  |  |  | 23.9 |  | 79 | 214 |  |  |  |
|  |  |  |  |  | 24.6 |  | 158 | 293 |  |  |  |
|  |  |  |  |  | 26.8 | 0 | 0 | 0 |  |  | 153 |
|  |  |  |  |  | 28.8 |  | 79 | 79 |  |  |  |
|  |  |  |  |  | 24.0 |  | 158 | 158 |  |  |  |
|  |  |  |  |  | 29.3 | 135 | 0 | 135 |  |  |  |
|  |  |  |  |  | 27.0 |  | 79 | 214 |  |  |  |
|  |  |  |  |  | 29.7 |  | 158 | 293 |  |  |  |
|  |  |  |  |  | 31.2 | 0 | 0 | 0 |  |  | 305 |
|  |  |  |  |  | 23.6 |  | 79 | 79 |  |  |  |
|  |  |  |  |  | 31.8 |  | 158 | 158 |  |  |  |
|  |  |  |  |  | 30.1 | 135 | 0 | 135 |  |  |  |
|  |  |  |  |  | 28.0 |  | 79 | 214 |  |  |  |
|  |  |  |  |  | 29.8 |  | 158 | 293 |  |  |  |
|  |  |  |  |  | 30.0 | 0 | 0 | 0 |  |  | 480 |
|  |  |  |  |  | 33.0 |  | 79 | 79 |  |  |  |
|  |  |  |  |  | 32.9 |  | 158 | 158 |  |  |  |
|  |  |  |  |  | 32.0 | 135 | 0 | 135 |  |  |  |
|  |  |  |  |  | 30.3 |  | 79 | 214 |  |  |  |
|  |  |  |  |  | 30.4 |  | 158 | 293 |  |  |  |
| Westwood | PioneerX | 20/11/2012 | 20/03/2013 | 8.2 | 27.3 | 135 | 158 | 293 | 20/11/2012 | 06/01/2013 | ETA* |
|  |  |  | 20/03/2013 | 5.5 | 28.4 |  |  |  |  |  |  |
|  |  |  | 08/04/2013 | 8.2 | 36.6 |  |  |  |  |  |  |
|  |  |  | 08/04/2013 | 5.5 | 40.7 |  |  |  |  |  |  |
|  |  |  | 15/04/2013 | 8.2 | 44.2 |  |  |  |  |  |  |
|  |  |  | 15/04/2013 | 5.5 | 47.2 |  |  |  |  |  |  |
|  | PacificX |  | 20/03/2013 | 8.2 | 29.9 |  |  |  |  |  |  |
|  |  |  | 20/03/2013 | 5.5 | 29.4 |  |  |  |  |  |  |
|  |  |  | 08/04/2013 | 8.2 | 36.0 |  |  |  |  |  |  |
|  |  |  | 08/04/2013 | 5.5 | 43.2 |  |  |  |  |  |  |
|  |  |  | 15/04/2013 | 8.2 | 44.1 |  |  |  |  |  |  |
|  |  |  | 15/04/2013 | 5.5 | 47.2 |  |  |  |  |  |  |
| P-farm | Pioneer 2307 | 04/11/2012 | 14/03/2013 | 7.7 | 31.3 | 216 | - | 216 | 04/11/2012 | - | ETA* |
|  |  |  | 14/03/2013 | 6.6 | 31.7 |  |  |  |  |  |  |
|  |  |  | 27/03/2013 | 7.2 | 38.2 |  |  |  |  |  |  |
|  |  |  | 27/03/2013 | 6.9 | 39.4 |  |  |  |  |  |  |
|  |  |  | 08/04/2013 | 7.4 | 45.0 |  |  |  |  |  |  |
|  |  |  | 08/04/2013 | 6.3 | 45.4 |  |  |  |  |  |  |

*ETA indicates irrigation follows evapotranspiration requirements.

**Supplementary Table 4.** Description of genotypic parameters and methods for parameter calibration in the model.

| **Parameter** | **Unit** | **Description** | **Process/es affected** | **Calibration method** |
| --- | --- | --- | --- | --- |
| *aX0* | proportion | Largest leaf position multiplier | leaf and stem weight | Parametrisation |
| *largestLeafParams* |  | Largest leaf area parameters | leaf and stem weight | Parametrisation |
| *leaf_app_r1* | °Cd | Leaf appearance rate coefficient 1 | final leaf number | Parametrisation |
| *leaf_app_r2* | °Cd | Leaf appearance rate coefficient 2 | final leaf number | Parametrisation |
| *leaf_app_r3* | °Cd | Leaf appearance rate coefficient 3 | final leaf number | Parametrisation |
| *leaf_no_rate_ch1* | leaves | Leaf number rate change coefficient 1 | final leaf number | Parametrisation |
| *leaf_no_rate_ch2* | leaves | Leaf number rate change coefficient 2 | final leaf number | Parametrisation |
| *tt_emerg_to_endjuv* | °Cd | Cumulative thermal time from emergence to end of juvenile phase (V6) | final leaf number  leaf and stem weight | Parametrisation |
| *tt_flower_to_maturity* | °Cd | Cumulative thermal time from flowering to physiological maturity (R6) | grain weight | Parametrisation |
| *frac_stem2flower* | 0-1 | Fraction of dry matter allocated to stem that goes to the developing head | cob and stem weight | Optimisation |
| stem_trans_frac | 0-1 | Fraction of stem dry matter potentially translocated to grain | stem and grain weight | Optimisation |
| *GNk* |  | Parameter related with the grain growth rate | grain number | Optimisation |
| *GNmaxCoef* | grains plant^-1^ | Potential number of grains per plant | grain number | Optimisation |
| *potKernelWt* | mg grain^-1^ | Potential grain weight | grain weight | Optimisation |
| *ph1* | h | Critical photoperiod 1 | final leaf number | Default |
| *ph2* | h | Critical photoperiod 2 | final leaf number | Default |
| *ph_s* | h | Photoperiod sensitivity | final leaf number | Default |
| *tt_flag_to_flower* | °Cd | Cumulative thermal time from flag leaf (VT) to flowering (R1) | grain number | Default |
| *tt_flower_to_start_grain* | °Cd | Cumulative thermal time from flowering (R1) to the start of grain filling (R2) | grain number | Default |
| *tt_maturity_to_ripe* | °Cd | Cumulative thermal time from physiological maturity (R6) to ripe | - | Default |

**Supplementary Table 5.** Statistical summary description for the absolute model prediction error (simulated – observed; S-O) and observed standard deviations (SD) by variable assessed in the model calibration. 25, 50 and 75% indicate the 0.25, 0.5 and 0.75 percentiles. The absolute model prediction error for harvest index is also showed.

| **Variable** | **Metric** | **unit** | **mean** | **minimum** | **25%** | **50%** | **75%** | **maximum** |
| --- | --- | --- | --- | --- | --- | --- | --- | --- |
| Leaf number | S-O | N leaves pl^-1^ | 0.94 | 0.02 | 0.25 | 0.67 | 1.32 | 4.09 |
|  | SD |  | 0.66 | 0.00 | 0.00 | 0.37 | 0.71 | 5.30 |
| Canopy green cover | S-O | 0-1 | 0.17 | 0.01 | 0.10 | 0.13 | 0.25 | 0.35 |
|  | SD |  | 0.04 | 0.00 | 0.01 | 0.03 | 0.05 | 0.10 |
| Aboveground biomass | S-O | gDM m^-2^ | 243 | 0 | 130 | 225 | 339 | 1026 |
|  | SD |  | 174 | 0 | 67 | 142 | 263 | 850 |
| Grain yield + cob weight | S-O | gDM m^-2^ | 133 | 3 | 77 | 121 | 171 | 563 |
|  | SD |  | 67 | 0 | 0 | 39 | 104 | 335 |
| Stover weight | S-O | gDM m^-2^ | 185 | 0 | 79 | 153 | 269 | 611 |
|  | SD |  | 136 | 0 | 41 | 110 | 205 | 850 |
| Leaf weight | S-O | gDM m^-2^ | 107 | 0 | 47 | 83 | 155 | 372 |
|  | SD |  | 48 | 0 | 8 | 33 | 78 | 415 |
| Stem weight | S-O | gDM m^-2^ | 77 | 1 | 29 | 55 | 91 | 381 |
|  | SD |  | 49 | 0 | 4 | 32 | 79 | 328 |
| Grain number | S-O | N grains m^-2^ | 786 | 43 | 462 | 694 | 984 | 2076 |
|  | SD |  | 784 | 85 | 562 | 1006 | 1006 | 1006 |
| Grain size | S-O | g 1000 grains^-1^ | 51 | 0 | 19 | 57 | 80 | 99 |
|  | SD |  | 66 | 17 | 48 | 85 | 85 | 85 |
| Harvest index | S-O | 0-1 | 0.040 | 0.001 | 0.013 | 0.037 | 0.061 | 0.114 |

**Supplementary Table 6.** Mean grain yield, biomass and harvest index (HI) for each combination of factors simulated across scenarios. DAS, days after sowing. Each value indicates the mean across 50 years.

| **Sowing date** | **Sowing density** | **Irrigation** | **Harvest date** | **Cultivar** | **N rate** | **Grain yield** | **Biomass** | **HI** |
| --- | --- | --- | --- | --- | --- | --- | --- | --- |
|  | *pl m^-2^* | *0-1* | *DAS* |  | *kg N ha^-1^* | *kg ha^-1^* | *kg ha^-1^* |  |
| 20-Oct | 9.3 | deficit | 125 | early | 250 | 12329 | 19166 | 0.64 |
| 20-Oct | 9.3 | deficit | 125 | early | 0 | 3784 | 8444 | 0.43 |
| 20-Oct | 9.3 | deficit | 125 | early | 125 | 10594 | 17319 | 0.61 |
| 20-Oct | 9.3 | deficit | 125 | late | 250 | 9298 | 23436 | 0.40 |
| 20-Oct | 9.3 | deficit | 125 | late | 0 | 452 | 7469 | 0.05 |
| 20-Oct | 9.3 | deficit | 125 | late | 125 | 9163 | 21638 | 0.43 |
| 20-Oct | 9.3 | deficit | 150 | early | 250 | 12271 | 19107 | 0.64 |
| 20-Oct | 9.3 | deficit | 150 | early | 0 | 3524 | 7977 | 0.43 |
| 20-Oct | 9.3 | deficit | 150 | early | 125 | 10538 | 17284 | 0.61 |
| 20-Oct | 9.3 | deficit | 150 | late | 250 | 13908 | 26259 | 0.53 |
| 20-Oct | 9.3 | deficit | 150 | late | 0 | 1860 | 7236 | 0.25 |
| 20-Oct | 9.3 | deficit | 150 | late | 125 | 12338 | 22005 | 0.56 |
| 20-Oct | 9.3 | deficit | 138 | early | 250 | 12318 | 19148 | 0.65 |
| 20-Oct | 9.3 | deficit | 138 | early | 0 | 3672 | 8229 | 0.43 |
| 20-Oct | 9.3 | deficit | 138 | early | 125 | 10537 | 17269 | 0.61 |
| 20-Oct | 9.3 | deficit | 138 | late | 250 | 12781 | 25714 | 0.50 |
| 20-Oct | 9.3 | deficit | 138 | late | 0 | 1172 | 7404 | 0.14 |
| 20-Oct | 9.3 | deficit | 138 | late | 125 | 11881 | 21960 | 0.54 |
| 20-Oct | 9.3 | rainfed | 125 | early | 250 | 9133 | 15014 | 0.60 |
| 20-Oct | 9.3 | rainfed | 125 | early | 0 | 3715 | 8386 | 0.43 |
| 20-Oct | 9.3 | rainfed | 125 | early | 125 | 8731 | 14839 | 0.58 |
| 20-Oct | 9.3 | rainfed | 125 | late | 250 | 6663 | 17396 | 0.39 |
| 20-Oct | 9.3 | rainfed | 125 | late | 0 | 391 | 7422 | 0.04 |
| 20-Oct | 9.3 | rainfed | 125 | late | 125 | 6561 | 16936 | 0.40 |
| 20-Oct | 9.3 | rainfed | 150 | early | 250 | 8926 | 14743 | 0.59 |
| 20-Oct | 9.3 | rainfed | 150 | early | 0 | 3476 | 7955 | 0.43 |
| 20-Oct | 9.3 | rainfed | 150 | early | 125 | 8792 | 14967 | 0.58 |
| 20-Oct | 9.3 | rainfed | 150 | late | 250 | 9893 | 19162 | 0.51 |
| 20-Oct | 9.3 | rainfed | 150 | late | 0 | 1834 | 7210 | 0.25 |
| 20-Oct | 9.3 | rainfed | 150 | late | 125 | 9535 | 17968 | 0.53 |
| 20-Oct | 9.3 | rainfed | 138 | early | 250 | 9020 | 14868 | 0.60 |
| 20-Oct | 9.3 | rainfed | 138 | early | 0 | 3605 | 8177 | 0.43 |
| 20-Oct | 9.3 | rainfed | 138 | early | 125 | 8764 | 14905 | 0.58 |
| 20-Oct | 9.3 | rainfed | 138 | late | 250 | 9011 | 18649 | 0.49 |
| 20-Oct | 9.3 | rainfed | 138 | late | 0 | 1108 | 7345 | 0.14 |
| 20-Oct | 9.3 | rainfed | 138 | late | 125 | 8804 | 17685 | 0.50 |
| 20-Oct | 9.3 | well-irri | 125 | early | 250 | 13340 | 21053 | 0.63 |
| 20-Oct | 9.3 | well-irri | 125 | early | 0 | 4140 | 9080 | 0.44 |
| 20-Oct | 9.3 | well-irri | 125 | early | 125 | 11341 | 18715 | 0.61 |
| 20-Oct | 9.3 | well-irri | 125 | late | 250 | 10029 | 25854 | 0.39 |
| 20-Oct | 9.3 | well-irri | 125 | late | 0 | 467 | 7832 | 0.05 |
| 20-Oct | 9.3 | well-irri | 125 | late | 125 | 9765 | 22923 | 0.43 |
| 20-Oct | 9.3 | well-irri | 150 | early | 250 | 13310 | 21019 | 0.63 |
| 20-Oct | 9.3 | well-irri | 150 | early | 0 | 3814 | 8454 | 0.44 |
| 20-Oct | 9.3 | well-irri | 150 | early | 125 | 11205 | 18561 | 0.60 |
| 20-Oct | 9.3 | well-irri | 150 | late | 250 | 14817 | 28271 | 0.53 |
| 20-Oct | 9.3 | well-irri | 150 | late | 0 | 1938 | 7597 | 0.25 |
| 20-Oct | 9.3 | well-irri | 150 | late | 125 | 12780 | 23116 | 0.55 |
| 20-Oct | 9.3 | well-irri | 138 | early | 250 | 13326 | 21030 | 0.63 |
| 20-Oct | 9.3 | well-irri | 138 | early | 0 | 3986 | 8774 | 0.44 |
| 20-Oct | 9.3 | well-irri | 138 | early | 125 | 11277 | 18643 | 0.60 |
| 20-Oct | 9.3 | well-irri | 138 | late | 250 | 13682 | 27902 | 0.49 |
| 20-Oct | 9.3 | well-irri | 138 | late | 0 | 1176 | 7742 | 0.14 |
| 20-Oct | 9.3 | well-irri | 138 | late | 125 | 12396 | 23119 | 0.54 |
| 20-Oct | 5.2 | deficit | 125 | early | 250 | 9663 | 16297 | 0.60 |
| 20-Oct | 5.2 | deficit | 125 | early | 0 | 5238 | 9629 | 0.54 |
| 20-Oct | 5.2 | deficit | 125 | early | 125 | 9453 | 15967 | 0.60 |
| 20-Oct | 5.2 | deficit | 125 | late | 250 | 6807 | 20399 | 0.34 |
| 20-Oct | 5.2 | deficit | 125 | late | 0 | 1916 | 8797 | 0.19 |
| 20-Oct | 5.2 | deficit | 125 | late | 125 | 6802 | 20218 | 0.34 |
| 20-Oct | 5.2 | deficit | 150 | early | 250 | 9648 | 16238 | 0.60 |
| 20-Oct | 5.2 | deficit | 150 | early | 0 | 4962 | 9236 | 0.53 |
| 20-Oct | 5.2 | deficit | 150 | early | 125 | 9369 | 15765 | 0.60 |
| 20-Oct | 5.2 | deficit | 150 | late | 250 | 10478 | 23850 | 0.44 |
| 20-Oct | 5.2 | deficit | 150 | late | 0 | 3199 | 8474 | 0.36 |
| 20-Oct | 5.2 | deficit | 150 | late | 125 | 10080 | 22008 | 0.46 |
| 20-Oct | 5.2 | deficit | 138 | early | 250 | 9648 | 16267 | 0.60 |
| 20-Oct | 5.2 | deficit | 138 | early | 0 | 5106 | 9442 | 0.53 |
| 20-Oct | 5.2 | deficit | 138 | early | 125 | 9402 | 15848 | 0.60 |
| 20-Oct | 5.2 | deficit | 138 | late | 250 | 9446 | 22795 | 0.42 |
| 20-Oct | 5.2 | deficit | 138 | late | 0 | 2658 | 8551 | 0.29 |
| 20-Oct | 5.2 | deficit | 138 | late | 125 | 9313 | 21784 | 0.43 |
| 20-Oct | 5.2 | rainfed | 125 | early | 250 | 7867 | 13198 | 0.60 |
| 20-Oct | 5.2 | rainfed | 125 | early | 0 | 5204 | 9615 | 0.54 |
| 20-Oct | 5.2 | rainfed | 125 | early | 125 | 7773 | 13204 | 0.59 |
| 20-Oct | 5.2 | rainfed | 125 | late | 250 | 5422 | 15572 | 0.36 |
| 20-Oct | 5.2 | rainfed | 125 | late | 0 | 1667 | 8574 | 0.17 |
| 20-Oct | 5.2 | rainfed | 125 | late | 125 | 5426 | 15522 | 0.37 |
| 20-Oct | 5.2 | rainfed | 150 | early | 250 | 7603 | 12835 | 0.59 |
| 20-Oct | 5.2 | rainfed | 150 | early | 0 | 4998 | 9325 | 0.53 |
| 20-Oct | 5.2 | rainfed | 150 | early | 125 | 7651 | 13054 | 0.59 |
| 20-Oct | 5.2 | rainfed | 150 | late | 250 | 8266 | 17448 | 0.48 |
| 20-Oct | 5.2 | rainfed | 150 | late | 0 | 3074 | 8359 | 0.35 |
| 20-Oct | 5.2 | rainfed | 150 | late | 125 | 8152 | 17101 | 0.48 |
| 20-Oct | 5.2 | rainfed | 138 | early | 250 | 7634 | 12859 | 0.59 |
| 20-Oct | 5.2 | rainfed | 138 | early | 0 | 5106 | 9478 | 0.53 |
| 20-Oct | 5.2 | rainfed | 138 | early | 125 | 7657 | 13038 | 0.59 |
| 20-Oct | 5.2 | rainfed | 138 | late | 250 | 7463 | 16910 | 0.45 |
| 20-Oct | 5.2 | rainfed | 138 | late | 0 | 2509 | 8399 | 0.28 |
| 20-Oct | 5.2 | rainfed | 138 | late | 125 | 7358 | 16559 | 0.46 |
| 20-Oct | 5.2 | well-irri | 125 | early | 250 | 9969 | 17506 | 0.57 |
| 20-Oct | 5.2 | well-irri | 125 | early | 0 | 5758 | 10429 | 0.55 |
| 20-Oct | 5.2 | well-irri | 125 | early | 125 | 9762 | 16975 | 0.58 |
| 20-Oct | 5.2 | well-irri | 125 | late | 250 | 7127 | 22147 | 0.32 |
| 20-Oct | 5.2 | well-irri | 125 | late | 0 | 2325 | 9530 | 0.21 |
| 20-Oct | 5.2 | well-irri | 125 | late | 125 | 7119 | 21860 | 0.33 |
| 20-Oct | 5.2 | well-irri | 150 | early | 250 | 9956 | 17466 | 0.57 |
| 20-Oct | 5.2 | well-irri | 150 | early | 0 | 5382 | 9881 | 0.54 |
| 20-Oct | 5.2 | well-irri | 150 | early | 125 | 9694 | 16774 | 0.58 |
| 20-Oct | 5.2 | well-irri | 150 | late | 250 | 10916 | 25789 | 0.42 |
| 20-Oct | 5.2 | well-irri | 150 | late | 0 | 3802 | 9258 | 0.39 |
| 20-Oct | 5.2 | well-irri | 150 | late | 125 | 10485 | 23424 | 0.45 |
| 20-Oct | 5.2 | well-irri | 138 | early | 250 | 9962 | 17487 | 0.57 |
| 20-Oct | 5.2 | well-irri | 138 | early | 0 | 5574 | 10165 | 0.54 |
| 20-Oct | 5.2 | well-irri | 138 | early | 125 | 9734 | 16892 | 0.58 |
| 20-Oct | 5.2 | well-irri | 138 | late | 250 | 9873 | 24772 | 0.40 |
| 20-Oct | 5.2 | well-irri | 138 | late | 0 | 3206 | 9347 | 0.31 |
| 20-Oct | 5.2 | well-irri | 138 | late | 125 | 9705 | 23270 | 0.42 |
| 20-Oct | 7.2 | deficit | 125 | early | 250 | 11501 | 18120 | 0.64 |
| 20-Oct | 7.2 | deficit | 125 | early | 0 | 4444 | 9024 | 0.48 |
| 20-Oct | 7.2 | deficit | 125 | early | 125 | 10414 | 16766 | 0.62 |
| 20-Oct | 7.2 | deficit | 125 | late | 250 | 8280 | 22412 | 0.37 |
| 20-Oct | 7.2 | deficit | 125 | late | 0 | 905 | 8002 | 0.09 |
| 20-Oct | 7.2 | deficit | 125 | late | 125 | 8229 | 21589 | 0.39 |
| 20-Oct | 7.2 | deficit | 150 | early | 250 | 11431 | 18010 | 0.64 |
| 20-Oct | 7.2 | deficit | 150 | early | 0 | 4110 | 8517 | 0.47 |
| 20-Oct | 7.2 | deficit | 150 | early | 125 | 10342 | 16682 | 0.62 |
| 20-Oct | 7.2 | deficit | 150 | late | 250 | 12477 | 25723 | 0.49 |
| 20-Oct | 7.2 | deficit | 150 | late | 0 | 2407 | 7773 | 0.30 |
| 20-Oct | 7.2 | deficit | 150 | late | 125 | 12061 | 22406 | 0.54 |
| 20-Oct | 7.2 | deficit | 138 | early | 250 | 11483 | 18074 | 0.64 |
| 20-Oct | 7.2 | deficit | 138 | early | 0 | 4274 | 8778 | 0.47 |
| 20-Oct | 7.2 | deficit | 138 | early | 125 | 10366 | 16709 | 0.62 |
| 20-Oct | 7.2 | deficit | 138 | late | 250 | 11410 | 24934 | 0.46 |
| 20-Oct | 7.2 | deficit | 138 | late | 0 | 1696 | 7870 | 0.20 |
| 20-Oct | 7.2 | deficit | 138 | late | 125 | 11187 | 22345 | 0.50 |
| 20-Oct | 7.2 | rainfed | 125 | early | 250 | 8741 | 14306 | 0.61 |
| 20-Oct | 7.2 | rainfed | 125 | early | 0 | 4421 | 9040 | 0.48 |
| 20-Oct | 7.2 | rainfed | 125 | early | 125 | 8479 | 14203 | 0.59 |
| 20-Oct | 7.2 | rainfed | 125 | late | 250 | 6136 | 16656 | 0.38 |
| 20-Oct | 7.2 | rainfed | 125 | late | 0 | 791 | 7886 | 0.09 |
| 20-Oct | 7.2 | rainfed | 125 | late | 125 | 6131 | 16526 | 0.38 |
| 20-Oct | 7.2 | rainfed | 150 | early | 250 | 8520 | 14033 | 0.60 |
| 20-Oct | 7.2 | rainfed | 150 | early | 0 | 4133 | 8595 | 0.47 |
| 20-Oct | 7.2 | rainfed | 150 | early | 125 | 8422 | 14148 | 0.59 |
| 20-Oct | 7.2 | rainfed | 150 | late | 250 | 9275 | 18562 | 0.50 |
| 20-Oct | 7.2 | rainfed | 150 | late | 0 | 2308 | 7660 | 0.29 |
| 20-Oct | 7.2 | rainfed | 150 | late | 125 | 9130 | 17815 | 0.51 |
| 20-Oct | 7.2 | rainfed | 138 | early | 250 | 8583 | 14107 | 0.60 |
| 20-Oct | 7.2 | rainfed | 138 | early | 0 | 4291 | 8837 | 0.47 |
| 20-Oct | 7.2 | rainfed | 138 | early | 125 | 8459 | 14178 | 0.59 |
| 20-Oct | 7.2 | rainfed | 138 | late | 250 | 8442 | 18024 | 0.48 |
| 20-Oct | 7.2 | rainfed | 138 | late | 0 | 1574 | 7744 | 0.19 |
| 20-Oct | 7.2 | rainfed | 138 | late | 125 | 8304 | 17457 | 0.48 |
| 20-Oct | 7.2 | well-irri | 125 | early | 250 | 12213 | 19825 | 0.62 |
| 20-Oct | 7.2 | well-irri | 125 | early | 0 | 4964 | 9848 | 0.49 |
| 20-Oct | 7.2 | well-irri | 125 | early | 125 | 11129 | 18105 | 0.62 |
| 20-Oct | 7.2 | well-irri | 125 | late | 250 | 8819 | 24685 | 0.36 |
| 20-Oct | 7.2 | well-irri | 125 | late | 0 | 1086 | 8550 | 0.10 |
| 20-Oct | 7.2 | well-irri | 125 | late | 125 | 8770 | 23200 | 0.38 |
| 20-Oct | 7.2 | well-irri | 150 | early | 250 | 12229 | 19838 | 0.62 |
| 20-Oct | 7.2 | well-irri | 150 | early | 0 | 4509 | 9144 | 0.48 |
| 20-Oct | 7.2 | well-irri | 150 | early | 125 | 10984 | 17909 | 0.61 |
| 20-Oct | 7.2 | well-irri | 150 | late | 250 | 13223 | 28014 | 0.47 |
| 20-Oct | 7.2 | well-irri | 150 | late | 0 | 2587 | 8215 | 0.30 |
| 20-Oct | 7.2 | well-irri | 150 | late | 125 | 12723 | 23667 | 0.54 |
| 20-Oct | 7.2 | well-irri | 138 | early | 250 | 12197 | 19810 | 0.62 |
| 20-Oct | 7.2 | well-irri | 138 | early | 0 | 4739 | 9503 | 0.48 |
| 20-Oct | 7.2 | well-irri | 138 | early | 125 | 11064 | 18017 | 0.61 |
| 20-Oct | 7.2 | well-irri | 138 | late | 250 | 12112 | 27248 | 0.45 |
| 20-Oct | 7.2 | well-irri | 138 | late | 0 | 1868 | 8369 | 0.20 |
| 20-Oct | 7.2 | well-irri | 138 | late | 125 | 11847 | 23671 | 0.50 |
| 3-Nov | 9.3 | deficit | 125 | early | 250 | 11918 | 18235 | 0.66 |
| 3-Nov | 9.3 | deficit | 125 | early | 0 | 3747 | 8197 | 0.44 |
| 3-Nov | 9.3 | deficit | 125 | early | 125 | 10415 | 16676 | 0.63 |
| 3-Nov | 9.3 | deficit | 125 | late | 250 | 10181 | 23630 | 0.43 |
| 3-Nov | 9.3 | deficit | 125 | late | 0 | 641 | 7351 | 0.07 |
| 3-Nov | 9.3 | deficit | 125 | late | 125 | 9846 | 21293 | 0.47 |
| 3-Nov | 9.3 | deficit | 150 | early | 250 | 11770 | 18097 | 0.65 |
| 3-Nov | 9.3 | deficit | 150 | early | 0 | 3501 | 7716 | 0.44 |
| 3-Nov | 9.3 | deficit | 150 | early | 125 | 10317 | 16595 | 0.62 |
| 3-Nov | 9.3 | deficit | 150 | late | 250 | 13929 | 25888 | 0.54 |
| 3-Nov | 9.3 | deficit | 150 | late | 0 | 1910 | 7165 | 0.26 |
| 3-Nov | 9.3 | deficit | 150 | late | 125 | 12311 | 21534 | 0.57 |
| 3-Nov | 9.3 | deficit | 138 | early | 250 | 11854 | 18177 | 0.65 |
| 3-Nov | 9.3 | deficit | 138 | early | 0 | 3623 | 7960 | 0.44 |
| 3-Nov | 9.3 | deficit | 138 | early | 125 | 10360 | 16632 | 0.62 |
| 3-Nov | 9.3 | deficit | 138 | late | 250 | 13161 | 25581 | 0.52 |
| 3-Nov | 9.3 | deficit | 138 | late | 0 | 1287 | 7292 | 0.16 |
| 3-Nov | 9.3 | deficit | 138 | late | 125 | 12050 | 21527 | 0.56 |
| 3-Nov | 9.3 | rainfed | 125 | early | 250 | 9233 | 14661 | 0.62 |
| 3-Nov | 9.3 | rainfed | 125 | early | 0 | 3711 | 8174 | 0.44 |
| 3-Nov | 9.3 | rainfed | 125 | early | 125 | 8851 | 14594 | 0.60 |
| 3-Nov | 9.3 | rainfed | 125 | late | 250 | 7453 | 17687 | 0.43 |
| 3-Nov | 9.3 | rainfed | 125 | late | 0 | 652 | 7351 | 0.07 |
| 3-Nov | 9.3 | rainfed | 125 | late | 125 | 7396 | 17233 | 0.44 |
| 3-Nov | 9.3 | rainfed | 150 | early | 250 | 8894 | 14228 | 0.61 |
| 3-Nov | 9.3 | rainfed | 150 | early | 0 | 3483 | 7735 | 0.44 |
| 3-Nov | 9.3 | rainfed | 150 | early | 125 | 8843 | 14625 | 0.60 |
| 3-Nov | 9.3 | rainfed | 150 | late | 250 | 10117 | 19003 | 0.53 |
| 3-Nov | 9.3 | rainfed | 150 | late | 0 | 1935 | 7163 | 0.26 |
| 3-Nov | 9.3 | rainfed | 150 | late | 125 | 9885 | 18062 | 0.54 |
| 3-Nov | 9.3 | rainfed | 138 | early | 250 | 9123 | 14528 | 0.62 |
| 3-Nov | 9.3 | rainfed | 138 | early | 0 | 3621 | 7994 | 0.44 |
| 3-Nov | 9.3 | rainfed | 138 | early | 125 | 8901 | 14710 | 0.60 |
| 3-Nov | 9.3 | rainfed | 138 | late | 250 | 9604 | 18950 | 0.51 |
| 3-Nov | 9.3 | rainfed | 138 | late | 0 | 1293 | 7282 | 0.16 |
| 3-Nov | 9.3 | rainfed | 138 | late | 125 | 9350 | 17805 | 0.52 |
| 3-Nov | 9.3 | well-irri | 125 | early | 250 | 12719 | 19856 | 0.64 |
| 3-Nov | 9.3 | well-irri | 125 | early | 0 | 4155 | 8907 | 0.45 |
| 3-Nov | 9.3 | well-irri | 125 | early | 125 | 11044 | 17935 | 0.62 |
| 3-Nov | 9.3 | well-irri | 125 | late | 250 | 10873 | 25745 | 0.42 |
| 3-Nov | 9.3 | well-irri | 125 | late | 0 | 602 | 7693 | 0.06 |
| 3-Nov | 9.3 | well-irri | 125 | late | 125 | 10421 | 22380 | 0.47 |
| 3-Nov | 9.3 | well-irri | 150 | early | 250 | 12731 | 19869 | 0.64 |
| 3-Nov | 9.3 | well-irri | 150 | early | 0 | 3847 | 8303 | 0.45 |
| 3-Nov | 9.3 | well-irri | 150 | early | 125 | 10896 | 17770 | 0.61 |
| 3-Nov | 9.3 | well-irri | 150 | late | 250 | 14770 | 27682 | 0.53 |
| 3-Nov | 9.3 | well-irri | 150 | late | 0 | 1984 | 7492 | 0.25 |
| 3-Nov | 9.3 | well-irri | 150 | late | 125 | 12669 | 22509 | 0.56 |
| 3-Nov | 9.3 | well-irri | 138 | early | 250 | 12722 | 19863 | 0.64 |
| 3-Nov | 9.3 | well-irri | 138 | early | 0 | 4018 | 8621 | 0.45 |
| 3-Nov | 9.3 | well-irri | 138 | early | 125 | 10973 | 17854 | 0.61 |
| 3-Nov | 9.3 | well-irri | 138 | late | 250 | 13987 | 27451 | 0.51 |
| 3-Nov | 9.3 | well-irri | 138 | late | 0 | 1292 | 7619 | 0.15 |
| 3-Nov | 9.3 | well-irri | 138 | late | 125 | 12468 | 22533 | 0.55 |
| 3-Nov | 5.2 | deficit | 125 | early | 250 | 9377 | 15464 | 0.61 |
| 3-Nov | 5.2 | deficit | 125 | early | 0 | 5115 | 9317 | 0.54 |
| 3-Nov | 5.2 | deficit | 125 | early | 125 | 9155 | 15098 | 0.61 |
| 3-Nov | 5.2 | deficit | 125 | late | 250 | 7413 | 20519 | 0.36 |
| 3-Nov | 5.2 | deficit | 125 | late | 0 | 2344 | 8802 | 0.23 |
| 3-Nov | 5.2 | deficit | 125 | late | 125 | 7401 | 20216 | 0.37 |
| 3-Nov | 5.2 | deficit | 150 | early | 250 | 9375 | 15464 | 0.61 |
| 3-Nov | 5.2 | deficit | 150 | early | 0 | 4836 | 8923 | 0.53 |
| 3-Nov | 5.2 | deficit | 150 | early | 125 | 9081 | 14948 | 0.61 |
| 3-Nov | 5.2 | deficit | 150 | late | 250 | 10493 | 23444 | 0.45 |
| 3-Nov | 5.2 | deficit | 150 | late | 0 | 3494 | 8576 | 0.39 |
| 3-Nov | 5.2 | deficit | 150 | late | 125 | 10099 | 21660 | 0.47 |
| 3-Nov | 5.2 | deficit | 138 | early | 250 | 9351 | 15417 | 0.61 |
| 3-Nov | 5.2 | deficit | 138 | early | 0 | 4991 | 9148 | 0.53 |
| 3-Nov | 5.2 | deficit | 138 | early | 125 | 9131 | 15041 | 0.61 |
| 3-Nov | 5.2 | deficit | 138 | late | 250 | 9740 | 22803 | 0.43 |
| 3-Nov | 5.2 | deficit | 138 | late | 0 | 3001 | 8593 | 0.32 |
| 3-Nov | 5.2 | deficit | 138 | late | 125 | 9568 | 21542 | 0.45 |
| 3-Nov | 5.2 | rainfed | 125 | early | 250 | 7798 | 12803 | 0.61 |
| 3-Nov | 5.2 | rainfed | 125 | early | 0 | 5109 | 9312 | 0.54 |
| 3-Nov | 5.2 | rainfed | 125 | early | 125 | 7802 | 12908 | 0.61 |
| 3-Nov | 5.2 | rainfed | 125 | late | 250 | 6012 | 15855 | 0.39 |
| 3-Nov | 5.2 | rainfed | 125 | late | 0 | 2054 | 8578 | 0.21 |
| 3-Nov | 5.2 | rainfed | 125 | late | 125 | 6007 | 15797 | 0.39 |
| 3-Nov | 5.2 | rainfed | 150 | early | 250 | 7581 | 12500 | 0.60 |
| 3-Nov | 5.2 | rainfed | 150 | early | 0 | 4884 | 9000 | 0.53 |
| 3-Nov | 5.2 | rainfed | 150 | early | 125 | 7709 | 12786 | 0.60 |
| 3-Nov | 5.2 | rainfed | 150 | late | 250 | 8289 | 17178 | 0.49 |
| 3-Nov | 5.2 | rainfed | 150 | late | 0 | 3342 | 8416 | 0.38 |
| 3-Nov | 5.2 | rainfed | 150 | late | 125 | 8282 | 17092 | 0.49 |
| 3-Nov | 5.2 | rainfed | 138 | early | 250 | 7678 | 12660 | 0.61 |
| 3-Nov | 5.2 | rainfed | 138 | early | 0 | 5014 | 9185 | 0.54 |
| 3-Nov | 5.2 | rainfed | 138 | early | 125 | 7728 | 12794 | 0.61 |
| 3-Nov | 5.2 | rainfed | 138 | late | 250 | 7801 | 17082 | 0.47 |
| 3-Nov | 5.2 | rainfed | 138 | late | 0 | 2856 | 8446 | 0.31 |
| 3-Nov | 5.2 | rainfed | 138 | late | 125 | 7750 | 16835 | 0.47 |
| 3-Nov | 5.2 | well-irri | 125 | early | 250 | 9720 | 16647 | 0.59 |
| 3-Nov | 5.2 | well-irri | 125 | early | 0 | 5605 | 10083 | 0.55 |
| 3-Nov | 5.2 | well-irri | 125 | early | 125 | 9562 | 16235 | 0.59 |
| 3-Nov | 5.2 | well-irri | 125 | late | 250 | 7741 | 22271 | 0.35 |
| 3-Nov | 5.2 | well-irri | 125 | late | 0 | 2637 | 9446 | 0.24 |
| 3-Nov | 5.2 | well-irri | 125 | late | 125 | 7722 | 21808 | 0.35 |
| 3-Nov | 5.2 | well-irri | 150 | early | 250 | 9708 | 16614 | 0.59 |
| 3-Nov | 5.2 | well-irri | 150 | early | 0 | 5253 | 9562 | 0.54 |
| 3-Nov | 5.2 | well-irri | 150 | early | 125 | 9512 | 16038 | 0.60 |
| 3-Nov | 5.2 | well-irri | 150 | late | 250 | 10907 | 25293 | 0.43 |
| 3-Nov | 5.2 | well-irri | 150 | late | 0 | 3854 | 9165 | 0.40 |
| 3-Nov | 5.2 | well-irri | 150 | late | 125 | 10480 | 23042 | 0.46 |
| 3-Nov | 5.2 | well-irri | 138 | early | 250 | 9711 | 16622 | 0.59 |
| 3-Nov | 5.2 | well-irri | 138 | early | 0 | 5436 | 9837 | 0.55 |
| 3-Nov | 5.2 | well-irri | 138 | early | 125 | 9541 | 16144 | 0.59 |
| 3-Nov | 5.2 | well-irri | 138 | late | 250 | 10146 | 24595 | 0.41 |
| 3-Nov | 5.2 | well-irri | 138 | late | 0 | 3393 | 9263 | 0.34 |
| 3-Nov | 5.2 | well-irri | 138 | late | 125 | 9939 | 22967 | 0.43 |
| 3-Nov | 7.2 | deficit | 125 | early | 250 | 11120 | 17189 | 0.65 |
| 3-Nov | 7.2 | deficit | 125 | early | 0 | 4350 | 8746 | 0.48 |
| 3-Nov | 7.2 | deficit | 125 | early | 125 | 10181 | 16081 | 0.63 |
| 3-Nov | 7.2 | deficit | 125 | late | 250 | 9061 | 22624 | 0.40 |
| 3-Nov | 7.2 | deficit | 125 | late | 0 | 1161 | 7939 | 0.12 |
| 3-Nov | 7.2 | deficit | 125 | late | 125 | 8968 | 21416 | 0.42 |
| 3-Nov | 7.2 | deficit | 150 | early | 250 | 11054 | 17106 | 0.65 |
| 3-Nov | 7.2 | deficit | 150 | early | 0 | 4089 | 8302 | 0.47 |
| 3-Nov | 7.2 | deficit | 150 | early | 125 | 10084 | 15966 | 0.63 |
| 3-Nov | 7.2 | deficit | 150 | late | 250 | 12524 | 25357 | 0.50 |
| 3-Nov | 7.2 | deficit | 150 | late | 0 | 2505 | 7711 | 0.31 |
| 3-Nov | 7.2 | deficit | 150 | late | 125 | 12134 | 21988 | 0.55 |
| 3-Nov | 7.2 | deficit | 138 | early | 250 | 11096 | 17166 | 0.65 |
| 3-Nov | 7.2 | deficit | 138 | early | 0 | 4229 | 8550 | 0.48 |
| 3-Nov | 7.2 | deficit | 138 | early | 125 | 10142 | 16032 | 0.63 |
| 3-Nov | 7.2 | deficit | 138 | late | 250 | 11781 | 24851 | 0.48 |
| 3-Nov | 7.2 | deficit | 138 | late | 0 | 1886 | 7796 | 0.22 |
| 3-Nov | 7.2 | deficit | 138 | late | 125 | 11505 | 21954 | 0.53 |
| 3-Nov | 7.2 | rainfed | 125 | early | 250 | 8755 | 13910 | 0.62 |
| 3-Nov | 7.2 | rainfed | 125 | early | 0 | 4339 | 8747 | 0.48 |
| 3-Nov | 7.2 | rainfed | 125 | early | 125 | 8575 | 13935 | 0.61 |
| 3-Nov | 7.2 | rainfed | 125 | late | 250 | 6882 | 16989 | 0.42 |
| 3-Nov | 7.2 | rainfed | 125 | late | 0 | 1189 | 7886 | 0.12 |
| 3-Nov | 7.2 | rainfed | 125 | late | 125 | 6870 | 16825 | 0.42 |
| 3-Nov | 7.2 | rainfed | 150 | early | 250 | 8516 | 13599 | 0.62 |
| 3-Nov | 7.2 | rainfed | 150 | early | 0 | 4108 | 8356 | 0.47 |
| 3-Nov | 7.2 | rainfed | 150 | early | 125 | 8489 | 13843 | 0.61 |
| 3-Nov | 7.2 | rainfed | 150 | late | 250 | 9450 | 18447 | 0.51 |
| 3-Nov | 7.2 | rainfed | 150 | late | 0 | 2511 | 7696 | 0.31 |
| 3-Nov | 7.2 | rainfed | 150 | late | 125 | 9346 | 17819 | 0.52 |
| 3-Nov | 7.2 | rainfed | 138 | early | 250 | 8711 | 13872 | 0.62 |
| 3-Nov | 7.2 | rainfed | 138 | early | 0 | 4228 | 8571 | 0.48 |
| 3-Nov | 7.2 | rainfed | 138 | early | 125 | 8573 | 13949 | 0.61 |
| 3-Nov | 7.2 | rainfed | 138 | late | 250 | 8919 | 18342 | 0.49 |
| 3-Nov | 7.2 | rainfed | 138 | late | 0 | 1917 | 7771 | 0.23 |
| 3-Nov | 7.2 | rainfed | 138 | late | 125 | 8729 | 17572 | 0.50 |
| 3-Nov | 7.2 | well-irri | 125 | early | 250 | 11819 | 18774 | 0.63 |
| 3-Nov | 7.2 | well-irri | 125 | early | 0 | 4935 | 9633 | 0.50 |
| 3-Nov | 7.2 | well-irri | 125 | early | 125 | 10831 | 17313 | 0.63 |
| 3-Nov | 7.2 | well-irri | 125 | late | 250 | 9570 | 24691 | 0.39 |
| 3-Nov | 7.2 | well-irri | 125 | late | 0 | 1266 | 8431 | 0.12 |
| 3-Nov | 7.2 | well-irri | 125 | late | 125 | 9506 | 22767 | 0.42 |
| 3-Nov | 7.2 | well-irri | 150 | early | 250 | 11780 | 18718 | 0.63 |
| 3-Nov | 7.2 | well-irri | 150 | early | 0 | 4512 | 8964 | 0.49 |
| 3-Nov | 7.2 | well-irri | 150 | early | 125 | 10692 | 17124 | 0.63 |
| 3-Nov | 7.2 | well-irri | 150 | late | 250 | 13184 | 27462 | 0.48 |
| 3-Nov | 7.2 | well-irri | 150 | late | 0 | 2648 | 8117 | 0.31 |
| 3-Nov | 7.2 | well-irri | 150 | late | 125 | 12695 | 23075 | 0.55 |
| 3-Nov | 7.2 | well-irri | 138 | early | 250 | 11798 | 18744 | 0.63 |
| 3-Nov | 7.2 | well-irri | 138 | early | 0 | 4715 | 9297 | 0.49 |
| 3-Nov | 7.2 | well-irri | 138 | early | 125 | 10768 | 17224 | 0.63 |
| 3-Nov | 7.2 | well-irri | 138 | late | 250 | 12402 | 26953 | 0.46 |
| 3-Nov | 7.2 | well-irri | 138 | late | 0 | 1990 | 8232 | 0.22 |
| 3-Nov | 7.2 | well-irri | 138 | late | 125 | 12092 | 23103 | 0.53 |
